# The role of transcription factors in the pathogenesis and therapeutic targeting of vascular diseases

**DOI:** 10.3389/fcvm.2024.1384294

**Published:** 2024-04-30

**Authors:** Poyi Hu, Yifan Du, Ying Xu, Ping Ye, Jiahong Xia

**Affiliations:** ^1^ Department of Cardiovascular Surgery, Union Hospital, Tongji Medical College, Huazhong University of Science and Technology, Wuhan, China; ^2^ Institute of Reproduction Health Research, Tongji Medical College, Huazhong University of Science and Technology, Wuhan, China; ^3^ Central Hospital of Wuhan, Huazhong University of Science and Technology, Wuhan, China

**Keywords:** transcription factors, vascular diseases, apoptosis, proliferation, inflammation, metabolism, stress

## Abstract

Transcription factors (TFs) constitute an essential component of epigenetic regulation. They contribute to the progression of vascular diseases by regulating epigenetic gene expression in several vascular diseases. Recently, numerous regulatory mechanisms related to vascular pathology, ranging from general TFs that are continuously activated to histiocyte-specific TFs that are activated under specific circumstances, have been studied. TFs participate in the progression of vascular-related diseases by epigenetically regulating vascular endothelial cells (VECs) and vascular smooth muscle cells (VSMCs). The Krüppel-like family (KLF) TF family is widely recognized as the foremost regulator of vascular diseases. KLF11 prevents aneurysm progression by inhibiting the apoptosis of VSMCs and enhancing their contractile function. The presence of KLF4, another crucial member, suppresses the progression of atherosclerosis (AS) and pulmonary hypertension by attenuating the formation of VSMCs-derived foam cells, ameliorating endothelial dysfunction, and inducing vasodilatory effects. However, the mechanism underlying the regulation of the progression of vascular-related diseases by TFs has remained elusive. The present study categorized the TFs involved in vascular diseases and their regulatory mechanisms to shed light on the potential pathogenesis of vascular diseases, and provide novel insights into their diagnosis and treatment.

## Introduction

TFs constitute a class of trans-acting factors that regulate the transcription of target genes (1). They execute this function by binding to specific DNA sequences of cis-acting elements (transcription factor-binding sites or transcription factor-binding structural domains) of target genes via hydrogen bonding (2). TFs are usually categorized into general TFs and tissue-specific TFs. General TFs bind with RNA polymerase II to form pre-initiation complex, which is responsible for the beginning of transcription (3). Within specialized cellular tissues, tissue-specific TFs are activated following the stimulation of extracellular inducers such as cytokines (4). The activated TFs participate in the regulation of cellular functions and the progression of diseases. Currently, TFs have been investigated in vascular diseases, and numerous TFs related to the progression of vascular diseases have been elucidated. For example, the pathogenesis of aneurysms involves the transcriptional regulation of genes associated with apoptosis, cellular senescence, inflammatory response, and oxidative stress (5, 6). TFs not only determine the expression of these genes, but the deletion of certain TF such as drosophila mothers against decapentaplegic (SMAD) 3 can also result in fatality without mice models (7). This highlights the importance of TFs in the occurrence and development of vascular diseases. TFs contribute to the progression of vascular-related diseases mostly by epigenetically regulating VECs and VSMCs. They are involved both in the basic transcriptional regulation of VECs and VSMCs and cell cycle regulation, cell development and differentiation, cellular inflammatory response, and metabolic changes (8). For instance, the forkhead box (Fox) TF family profoundly contributes to mammalian development, while the prevalence of vascular disease has risen among certain family members (9). Cell cycle regulation related TF FoxM1 promotes the proliferation of SMCs and ECs to intensify vascular remodeling (10). FoxO1 exacerbates the inflammatory response and promotes foam cell formation in response to cell stimulation, thereby exacerbating atherosclerotic lesions (11). The complete comprehension of the role of TFs in vascular diseases poses two significant challenges. Firstly, the understanding of the stimuli that trigger the pathogenic dysregulation of TFs is essential. For example, the construction of schematic models for pressure mechanical channels, shear stress types, and other physical parameters such as intima and media thickness at vessel bifurcation poses significant challenges in the study of vascular stress related to hemodynamic disorders (12, 13). The other aspect is that when regulation takes place in the form of a TF complex, it is often untoward to elucidate the downstream regulatory mechanisms of TF partners due to the presence of positive feedback loops (14). The primary therapeutic challenge lies in the design of small molecule drugs that can effectively bind to and inhibit the functional domain of TFs, thereby preventing pathogenic TFs' DNA binding domains from interacting with target genes. Meanwhile, the inhibition of both the catalytic domain and the protein binding domain by small molecule drugs should be taken into consideration simultaneously (15). In the present study, we summarize the TFs involved in vascular-related diseases and elucidate their cellular and molecular mechanisms in VSMCs, VECs, inflammation, and metabolism to provide novel diagnostic and therapeutic advantages in the future.

## TFs in vascular biology

In addition to capillaries, a vessel wall is composed of an intima, a tunica media, and an adventitia (16). Numerous TFs are involved in the transcriptional regulation of several disease-causing genes, especially during the vascular physiological disruption caused by different pathological factors and the progression of vascular diseases. Important cellular functions regulated by TFs in vascular diseases include apoptosis, proliferation, migration, aging, phenotypic switching of VSMCs, inflammation, stress, and metabolism. The investigation of TFs exemplifies the intricate nature of vascular biology. VSMCs constitute the tunica media of blood vessel walls, contributing to their structural integrity and elasticity (17). TF p53 plays a crucial role in the regulation of apoptosis, cell proliferation, and cell senescence, which is intricately associated with VSMCs-related vascular biology (18). Physiologically, the acetylation of p53 mediated by p300 in normal arteries forms the foundation for an appropriate stress response (19). The expression of p53 in vascular cells is typically low; however, p53 accumulates in aorta exacerbates apoptosis of aortic SMCs (20, 21). On the other hand, the increased expression of p53 hinders neointima formation by suppressing the proliferation of VSMCs, a process mediated by p21, while promoting cell senescence (22, 23). For VSMCs, the senescence-associated phenotype is affected by the pattern of glucose metabolism (24). Approximately 30% of ATP source of normal VSMCs depends on aerobic pathways, while HIF-1α is relatively high expressed (25, 26). When vascular injury occurs, VSMCs will undergo phenotypic switching from “contractile” to “synthetic” driven by metabolic pattern change, wherein TF hypoxia-inducing factor (HIF)-1α stimulates the shift of vascular cells towards increased reliance on anaerobic glycolysis (27). VECs form the tunica intima of blood vessel walls and serve as the primary defense component against the hemodynamic pressure (28). The SRY-related HMG-box (Sox) TF family associated with embryonic development and cell fate has a profound influence on the regulation of VECs-related cellular processes (29). Phenotypic shift-related TF Sox4, which is responsible for the endothelial-mesenchymal transition, not only contributes to the invasion of cancer cells, but also alters the fate of VECs and results in endothelial dysfunction when triggered by abnormal lipid metabolism and shear stress (30). While the ECs-specific TF Sox17, an essential member of the Sox family, maintains the normal proliferation extent of VECs (31). The latest research has demonstrated that the upregulation of specific TFs alters the ECs niches. Overexpression of TFs SoxF, Ets, and NHR induces ectopic ECs that support and recruit hematopoietic stem/progenitor cells, which normally tend to be stationary under the promotion of VECs (32, 33). These studies will better understand the effect of vascular niche on homeostasis and regeneration of blood stem cells. Meanwhile, the researchers proposed the experimental approach of “transcription factor cocktail”, wherein these three TFs were overexpressed in combination to investigate their impact on the vascular niche. Therefore, the extensive exploration of applying this concept in the presence of interactions between TFs is highly warranted. Chronic inflammatory infiltration constitutes an important step in the pathogenesis of vascular diseases, and the role of TFs such as the KLFs and nuclear factor (NF)-κB are being extensively investigated in vascular inflammation (34–36). KLFs maintains homeostasis of the vascular wall by regulating apoptosis, cell proliferation and differentiation, as well as inflammation through the involvement of co-regulators such as CREB-binding protein (CBP), histone deacetylases and p300/CBP-associated factor (37). The homeostasis regulation of VECs heavily relies on KLF2, which not only exerts an anti-inflammatory effect by up-regulating anti-inflammatory genes such as *PPAP2B* and activating endothelial nitric oxide synthase (eNOS) to suppress the expression of pro-inflammatory cytokines but also plays a crucial role in inhibiting thrombosis and angiogenesis (38–41). The activation of NF-κB triggers an inflammatory response characterized by the up-regulation of anti-apoptotic genes, secretion of inflammatory cytokines, and expression of adhesion molecules, which facilitates the recruitment of leukocytes to the response region (42). The majority of vascular NF-κB-related signaling molecules are existed in VECs, primarily involved in adhesion and coagulation processes when activated mainly by TNF-α and thrombin, while simultaneously compromising the barrier function (43, 44). Metabolisms play crucial roles in vascular physiology, with HIF-1α being an integral component of these processes (45). The key processes regulated by HIF-1α encompass angiogenesis, cell proliferation/survival, glucose metabolism, as well as iron metabolism (46). When cells experience hypoxia, HIF-1α stimulates the upregulation of glycolysis-related enzymes and facilitates glucose transportation through several glucose transporters (47). The upregulation of glycolysis not only facilitates vascular remodeling, but also expedites senescence-associated phenotypic transformation and compromises VSMCs function (48). Another isoform, HIF-2α, which is linked to insulin resistance induced by hyperlipidemia and exerts multiple mechanisms in regulating lipid metabolism, including promoting ceramide-mediated cholesterol clearance to exert an anti-atherosclerotic effect (49).

## Aneurysm-related TFs

Aneurysm refers to the irreversible dilation of the arterial wall (6). Fragmentation of elastin and weakening of the tunica media of the arterial wall constitutes the most significant pathologic features of aneurysms (50). Loss of elastic fibers is an early pathological change in aneurysms. The arterial wall initiates repair mechanisms in the form of collagen deposition as the disease progresses, whereas the determining factor for aneurysm rupture is collagen degradation, which is attributed to the imbalance between collagen synthesis and degradation (51, 52). Other pathologic features of aneurysms include loss of VSMCs, recruitment and infiltration of inflammatory cells such as lymphocytes and monocytes, and intraluminal thrombus (53–55). The lethality of ruptured aneurysms reaches 94% (56). Therefore, a comprehensive understanding of their pathogenesis is urgently required, and the transcriptional regulation of causative genes could be an important link in related studies.

The most decisive pathological changes in the progression of aneurysms are apoptosis and dysfunction of VSMCs in the tunica media, accompanied by the rupture of elastic fibers and collagen deposition, thus weakening the tunica media of the arterial wall and further dilatating or rupturing the arterial wall (53). While the dysfunctions exhibited by VSMCs encompass impaired contractile function and a phenotypic switching towards senescence and synthetic secretion. Salmon et al. reported upregulated expression of ZFP148, an apoptosis-related TF, in VSMCs of abdominal aortic aneurysm (AAA). ZFP148 activates the p21 and Bak promoters to inhibit the proliferation of VSMCs and induce apoptosis in VSMCs. In addition, they confirmed that ZFP148 inhibited the expression of SM22a and SM-actin, and caused VSMCs to lose function by suppressing the expression of SM22a and SM-actin (57). Similarly, Lu et al. demonstrated that the autophagy-related molecule TFEB activates the promoter of the apoptosis suppressor gene *Bcl2* to inhibit apoptosis in VSMCs and prevent the formation of AAA (58). The application of TFEB agonists trappsol cyclo has been observed in phase II clinical trials conducted for the treatment of Niemann-Pick Type C disease and has a anticipating potential in the treatment of AAA (59). The metabolic pattern of VSMCs is intricately related to the normal maintenance of their cellular functions, and alterations in the metabolic pattern of VSMCs stimulated by different pathogenic factors predispose them to unfavorable cellular phenotypes (24). Gao et al. reported that the deletion of p53, a senescence-associated TF, inhibited the mRNA and protein levels of *Sco2* and SCO2, respectively, thereby inactivating the transport chain complex IV. It induces a metabolic paradigm shift in VSMCs with reduced levels of oxidative phosphorylation and increased levels of glycolysis, causing the senescence of VSMCs and exacerbating AAA under energy limitation (60). Meanwhile, the increased expression of p53 in the aorta tissue induced the apoptosis of VSMCs by promoting the downstream apoptosis gene *Bax*, and as a response to the injury process that the degeneration lies in tunica media, the proliferation level in VSMCs was relatively elevated (21). Decreased contractile function of VSMCs is an important manifestation of VSMC dysfunction. Gong et al. reported that TF SMAD3 deficiency impairs the transforming growth factor (TGF)-β pathway, causing defective differentiation of VSMCs and decreased contractile function, accompanied by reduced elastin fibers synthesis and elevated collagen fiber deposition, and ultimately causing thoracic aortic aneurysm (61). The phenotypic transition of VSMCs in aneurysms is largely from a static “contractile” phenotype to a “synthetic” phenotype with proliferative, migratory, and pro-inflammatory properties (62). Chakraborty et al. reported that IRF3, a pro-inflammatory TF, which functions as a link in the STING (stimulator of interferon genes) pathway induces a shift from a contractile phenotype to a proliferative, extracellular matrix (ECM) production and an inflammatory phenotype in VSMCs following dsDNA stimulation, causing AAA (63). Zhao et al. reported that TF XBP1 was up-regulated in the AAA model and that it directly bound to the FoxO4 promoter to maintain the contractile phenotype of VSMCs, whereas the inhibition of their interactions induced a shift toward a pro-inflammatory and proteolytic phenotype of VSMCs (64). The migration of VSMCs is disadvantageous to the tunica media. Arif et al. reported that AP-1, which is responsible for regulating matrix metalloproteinases (MMPs), inhibits IL-1β-mediated migration of aortic VSMCs to delay aneurysm progression in mice with Marfan syndrome (65).

VECs constitute the first line of defense against the impact of blood flow, and their dysfunction is an important feature in the early progression of aneurysm. Continued exposure of VECs to excessive shear stress generates numerous reactive oxygen species (ROS), thus activating apoptosis- and inflammation-related pathways, producing endothelium-associated pathogenic cytokines such as inducible nitric oxide synthase, intercellular cell adhesion molecule (ICAM), and vascular cell-adhesion molecule (VCAM), and recruiting chemotaxis-induced macrophages recruitment and infiltrating MMPs, followed by complete collapse of the intima and dilatation of the vascular wall (66–68). Luo et al. reported that the recruitment of histone deacetylase 1-nucleosome remodeling and deacetylase complex by TF ZEB2 in VECs inhibits the cystathionine γ lyase/hydrogen sulfide system, inducing endothelial cell endoplasmic reticulum (ER) stress and exacerbating AAA (69). The administration of entinostat, which intercepts ZEB2-HDAC1 complex, effectively mitigates ER stress induced by the p-ERK pathway and restores the S-sulfhydration level of protein disulfide isomerase, demonstrating its potential for treating AAA. Lee et al. reported that the deletion of vascular differentiation, neogenesis-related TF SOX17 in VECs exacerbated intracranial aneurysm (IA). Deletion of *Sox17* up-regulates the cell cycle inhibitory molecules p15^INK4b^ and p16^INK4a^ and inhibits the proliferation of VECs, thereby promoting the progression of IA under chronic stress stimuli (70). The imbalance of ROS metabolism constitutes the primary factor in endothelial dysfunction (71). Zhao et al. reported that the downregulation of the vasoprotective TF E2F1-associated Rb/E2f1/Dhfr signaling pathway in VECs induces eNOS uncoupling-mediated ROS production, thereby elevating the incidence of AAA in AngII-induced hypertensive mice (72). Aoki et al. demonstrated that the COX-2-PGE2-EP2 signaling is up-regulated in patients with IA. This mechanism promotes NF-κB-mediated MCP-1 expression, exacerbating chronic inflammation of endothelial cells caused by excessive shear stress under hemodynamic shock and facilitating IA progression (73). Hamann et al. reported that the oxidative stress (OS)-related molecule CD163 is up-regulated in AAA, accompanied by up-regulation of the anti-OS TF NRF2 and its mediated HO-1, NQO-1, revealing the potential value of the CD163/NRF2-related pathway in the diagnosis of AAA (74). NF-κB is a crucial inflammation-associated TF in VECs, mediating localized chronic inflammation as well as neovascularization (75). Saito et al. inhibited NF-κB signaling in VECs and noted a substantial downregulation of the expression of SMC proliferation-related cytokines such as platelet derived growth factor subunit B (PDGF-B), ICAM-1 and VCAM-1, which subsequently suppressed macrophage-associated inflammation and OS, thereby inhibiting the generation of aortic aneurysms (76). Loss of function in VECs is usually accompanied by altered functions in VSMCs (77). For example, Zhao et al. observed cell differentiation and reported that apoptosis-related protective TF KLF11 is down-regulated in VECs of AAA mice. KLF11 not only inhibits inflammation and MMP expression in VECs but also binds to the Nox2 promoter to inhibit its mediated OS in VECs and delay AAA progression. In addition, KLF11 expression in VECs can maintain the contractile phenotype of VSMCs and inhibit VSMC apoptosis (78). An important step in inflammatory infiltration and thrombosis is the adhesion of VECs (79). Shin et al. reported the up-regulation of Egr-1, a pro-inflammatory, pro-adhesive, and pro-thrombotic TF in human AAA. The overexpression of Egr-1 in VECs up-regulates the expression of inflammatory chemokines such as MCP-1, MIP-1β, and ICAM-1, and tissue factors, thereby contributing to enhanced monocyte adhesion and thrombosis (80).

Inflammatory infiltrates are a hallmark of aneurysmal diseases. Inflammatory cells recruit and infiltrate largely myeloid cells and T lymphocytes. Different cytokines secreted by these inflammatory cells contribute to aneurysm progression. Cytokines associated with T-lymphocytes include interferon-γ, tumor necrosis factor (TNF)-α, and IL-17A, whereas those associated with myeloid cells are IL-1β, IL-6, and TGF-β. These cytokines influence the progression of aneurysms in macrophages. These cytokines affect the recruitment and infiltration of macrophages, neutrophils, and T cells in arterial tissues, ultimately destroying the survival of smooth muscle cells or endothelial cells, integrity of elastic fibers, and extracellular matrix, thereby exacerbating the progression of aneurysms (81, 82). The activation of the MAPK pathway is accompanied by several inflammatory responses, such as ERK-mediated expression of inflammatory factors, such as TNF and IL-1β, activation of NF-κB signaling, JNK-mediated macrophage M1 polarization, and pro-inflammatory cytokine expression mediated by the p38α/MK2 pathway (83). Martorell et al. reported that VDR, a TF associated with anti-inflammation and anti-proliferation, interacts with RXRα and inhibits the activation of MAPK pathway, VEGF expression, and ECM degradation in VECs, exhibiting anti-inflammatory and anti-neovascularization effects, thereby inhibiting AAA progression (84). In human clinical trials, the VDR activator calcitriol exhibited minimal toxicity at a dosage of 38ug/d; however, its efficacy in inhibiting MAPK pathway activation for the treatment of AAA remains unknown (85). Gao et al. reported that MKL1 expression was upregulated in AA. The deletion of MKL1 in VSMCs reduces the p38MAPK activity and inhibits vascular inflammation and senescence-related phenotypes, thereby preventing AA progression (86). Infiltrating T cells in the arterial wall of the aneurysmal tissue are predominantly CD4^+^T cells (87). Ye et al. reported that SMAD3-deficient mice spontaneously generated AA or aortic coarctation that ruptured and died, and the pathology involved chronic inflammation infiltrated by CD4^+^T cells throughout the aneurysm. In addition, the secretion of GM-CSF by cells caused elevated expression of MMPs, thus contributing to the progression of aneurysm (7). Another feature of CD4^+^T cells is their high secretion of IL-17, the signature cytokine of Th17 cells, which is responsible for inflammatory cell recruitment in the aorta (87). Romain et al. reported reduced IL-17 production and plasma IL-17 concentration in STAT3 signaling-deficient T cells, which delayed AAA progression in mice (88). Studies related to macrophage infiltration in aneurysms are intricately linked to the macrophage polarization. Increased M1 polarization of infiltrating macrophages suggests a higher incidence of aneurysm, faster progression, and a greater risk of rupture (89). A study by Han et al. demonstrated that Axl in macrophages mediates the up-regulation of the STAT1/HIF-1α pathway, causing M1 polarization and inducing a pro-inflammatory phenotype. The investigators reported that increased infiltration of M1-polarized macrophages at the lesion site led to a higher risk of IA rupture (90, 91). Axl and STAT1 phosphorylation inhibitor R248 significantly inhibited M1 polarization, which is promising in preventing the progression and rupture of IA.

**Figure 1:**
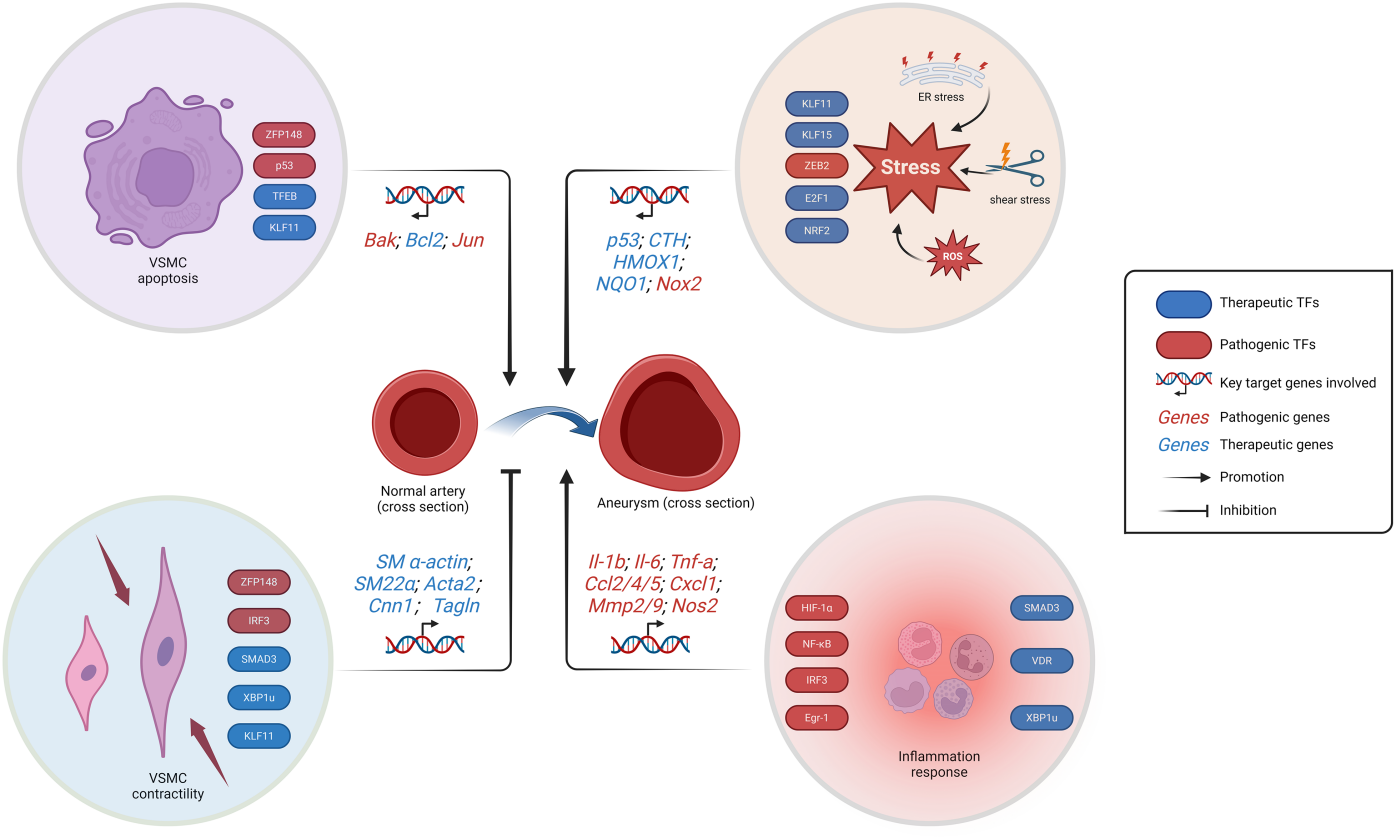
Schematic representation of the regulation of aneurysm-related TFs. The progression of aneurysms is facilitated by the loss of VSMCs, inflammation response, and stress. While the preservation of systolic function of VSMCs prevents aneurysm progression. The stress response encompasses endoplasmic reticulum stress, ROS generation, and vascular endothelial shear stress. VSMC, vascular smooth muscle cells; ER, endoplasmic reticulum; ROS, reactive oxygen species. Created with BioRender.com.

The innate immune response will continue to be a prominent area of research in the future, given the extensive repertoire of TFs associated with inflammatory response. Neutrophils play a pivotal role in the innate immune response, and neutrophil extracellular traps (NETs) have garnered increasing attention as a crucial pathological process in aneurysm-related research (92, 93). However, there remains a dearth of research on TFs in this context. The process of neutrophils from activation to death evolved rapidly, implying that the regulated release of NETs by neutrophils may involve various TFs such as ATF-2, TCF and NF-AT by transcriptome analysis and NETosis assay (94). In conclusion, TFs are involved in the regulation of many signaling pathways mainly related to apoptosis, stress and cell function maintenance in VSMCs and VECs, which are particularly important for aneurysms, and also affect the progression of aneurysms through the regulation of inflammatory response (Figure 1).

## Atherosclerosis-related TFs

AS lesions are known to originate in the intima. The primary pathologic changes in the early stages of AS include the generation of intimal foam cells and accumulation of ECM, causing dilatation of the intima. The progression of AS is exacerbated by intimal inflammation, weakened cellular burial, generation of an intimal fibrous cap, and stiffening of the vessel wall. Eventually, the intimal surface erodes, ruptures, and generates an intraluminal thrombus, causing life-threatening gradual luminal obstruction or even occlusion (95, 96).

The generation of foam cells constitutes a characteristic pathological change in AS. The accumulation of foam cells, containing lipid droplets, is the primary contributor to intimal thickening in the early stages of the disease. Foam cells are derived from macrophages maturing from circulating monocytes and smooth muscle cells migrating from the tunica media. As the disease progresses, the toxic effects of ox-LDL on endothelial cells and other cells in the microenvironment of the lesion cause necrosis and disintegration of foam cells, leading to atheromatous necrotic material within the plaque (97, 98). The NFAT TF family functions as a blocking target of FK506 and cyclosporine A (99). Liu et al. reported that the deletion of macrophage NFATc3 inhibits miR-204 expression and enhances scavenger receptor SR-A/CD36-mediated uptake of lipoproteins, thus promoting the generation of foam cells. Moreover, the scavenger receptor is an important pathway of macrophages for internalizing ox-LDL (100). Ma et al. reported that enhanced KLF4 expression and nuclear translocation inhibit lipid deposition in peripheral macrophages and VSMCs by down-regulating SR-A, which is required for M2 polarization of peripheral macrophages. In addition, this regulatory pathway suppresses the generation of foam cells by decreasing cholesterol uptake (101). IL-1β response is a crucial inflammatory pathway of foam cells involved in AS progression, which promotes the expansion of AS lesion areas along with PDGF, TNF, and M-CSF (95). Zhang et al. reported that the lack of mTORC2 in macrophages significantly increased FoxO1-mediated IL-1β response and aggravated AS progression (11). Mature macrophages are important sources of foam cells. Erbilgin et al. reported that a deficiency of Zhx2 in macrophages promoted macrophage apoptosis, inhibited macrophage M1 polarization, and stimulated M2 polarization. Zhx2 deficiency reduced both macrophage-derived foam cells and attenuated M1 macrophage-associated inflammation, thereby delaying AS progression. In addition, chromatin immunoprecipitation followed by sequencing (ChIP-seq) analysis revealed the apoptotic genes *Jun* and *Bcl6* as direct targets of Zhx2 (102).

In the early stage of AS, mesangial VSMCs migrated toward the intima to form resident SMCs. They undergo a phenotypic transition with the progression of AS, which is characterized by an increased proliferative capacity, decreased contractility, and diffuse thickening of the intima due to the massive secretion of ECM. Migrating VSMCs are not only involved in the generation of foam cells and necrotic core but also exhibit a macrophage-like inflammatory phenotype (95, 103, 104). Cheng et al. reported that the deletion of ZEB2, an epithelial-mesenchymal transition (EMT)-associated TF in VSMCs, inhibited TGF-β and Notch signaling, causing a reduced transition of VSMCs to a fibroblast-like myoblast phenotype and an accelerated transition to a chondroblast-like myoblast phenotype as well as disruption of the fibrous cap formation, causing a higher risk of AS plaques (105). Ledard et al. revealed the pathogenic function of Slug, another EMT-related TF, in AS. PDGF-BB has been reported to induce the expression of NF-κB and mTOR to promote the proliferation and migration of VSMCs, displaying potential AS pathogenicity (106). Researchers have reported the involvement of PDGF-BB in thrombosis, and its inhibition of Slug degradation via the ubiquitin-proteasome pathway greatly increased the expression of Slug. In addition, PDGF-BB down-regulates ABCA1 and ABCG1, cholesterol efflux-related genes in VSMCs, as well as induces the Slug-dependent switch to the inflammatory phenotype in VSMCs. These results suggest that Slug promotes thrombosis and induces plaque destabilization in AS (107). Lee et al. studied the proliferation of VSMCs and found that the endocrine nuclear receptor PR reduces the mRNA levels of the cell cycle proteins CCNA and CCNE, thereby inhibiting the proliferation of VSMCs. In addition, lower mortality rates have been reported in premenopausal women with AS as well as in those taking estrogen and progesterone (108–110).

Activation of the inflammatory response in VECs reduces the stability of plaques by proteolytic modification of ECM components. However, unstable plaques eventually disintegrate, leading to thrombosis and thus vascular occlusion, such that the modulation of the inflammatory response influences a patient's prognosis. Whether it is adhesion-associated molecules, ox-LDL, chemokines, or other inflammatory cytokines, NF-κB regulates the activation of endothelial cell inflammation (77). Karunakaran et al. reported that the inhibition of RIPK1 in VECs in response to inflammatory stimuli prevents NF-κB translocation to the nucleus, thereby inhibiting IL-1β signaling as well as monocyte adhesion (111). Jia et al. studied OS-associated inflammation and demonstrated that the expression of BACH1, an OS-associated TF, was up-regulated in AS plaques. Depletion of BACH1 in VECs inhibited the expression of adhesion molecules, thereby preventing monocyte adhesion and inflammatory cytokine expression. In addition, BACH1 co-localizes with YAP, a molecule associated with cytomechanical signaling and endothelial cell activation, and activates YAP transcription and co-promotes vascular inflammation (14). In protective TFs, Luo et al. reported that TF KLF2 activates the UCP2 promoter in VECs, and that deletion of UCP2 in VECs displayed a pro-inflammatory, pro-fibrotic phenotype, with increased collagen deposition that accelerated AS progression. In addition, UCP2 mediates Akt/FoxO1 inhibition through AMPK phosphorylation and alleviates endothelial inflammation (112). Increased production of MCP-1 in VECs directly leads to enhanced monocyte recruitment; for instance, Subramanian et al. analyzed an AngII-induced LDL-R knockout murine model of AS and demonstrated that the deletion of PPAR-γ increased MCP-1 production and exacerbated AS (113).

TFs related to lipid metabolism and microenvironmental homeostasis in AS have been extensively studied. Cholesterol homeostasis is balanced between the uptake/synthesis, efflux, and esterification, and excess cholesterol is transferred by LDL into the circulation and deposited in the arteries, thereby causing AS (114). Zhang et al. reported that exogenous administration of ceramide in high-fat-fed APOE knockout mice increased hepatic and plasma cholesterol levels. HIF-2α in adipocytes activates the ACER2 promoter, accelerating ceramide degradation and removing hepatic cholesterol, thereby preventing AS progression (49). Enhanced autophagic effects in macrophages not only reduce the number of foam cells but also eliminate lipids and control the progression of inflammation. Tao et al. reported that SR-B1, a scavenger receptor associated with autophagy-lysosome, activates PPAR-α, mediates TFEB nuclear translocation, and up-regulates autophagy effector molecules, VPS34, LC3, and ATGs, in macrophages (115). Caveolin-1 enhances autophagy, promotes lipid penetration in AS, and improves cholesterol metabolism and inflammatory response in AS (116). Yuan et al. reported that curcumin inhibits the nuclear translocation of SREBP-1, a cholesterol homeostasis-associated TF, and increases caveolin-1 expression in the aortic wall of APOE knockout mice. Similarly, curcumin improves lipid levels and prevents cholesterol accumulation in VSMCs (117). Diminished efferocytosis of macrophages prevents timely clearance of dead cells from the intima, causing increased intimal loading. Edsfeldt et al. reported that the expression of IRF5, a proinflammatory macrophage-associated TF, and CD11c, a monocyte marker, are associated with an unstable plaque-associated phenotype in human carotid plaques. IRF5 accelerates macrophage switching to a pro-inflammatory phenotype, thereby exacerbating plaque inflammatory infiltration and increasing necrotic core size by impairing macrophage efferocytosis, causing an increased risk of rupture in AS plaques (118). We have described in the previous section the mechanisms involved in shear stress in aneurysms, and the stabilization and degree of shear stress are intricately related to the promotion or inhibition of AS (119). Cheng et al. reported that cell development and differentiation-associated TF SOX4 expression are up-regulated in atherosclerotic aorta. Up-regulation of SOX4 in VECs accelerates the phenotypic shift of endothelial cells to mesenchymal-like endothelial cells, causing dysfunction of VECs and exacerbating AS progression. Furthermore, in contrast to steady shear stress, which exerts anti-AS effects in arterial blood flow, they reported that oscillatory shear stress up-regulated SOX4 and promoted AS progression (30). The activation of heat shock proteins (HSPs) in AS lesions not only damages VECs and activates macrophages but also influences lipid metabolism (120). Krishnamurthy et al. reported that HSF-1, an HSP regulatory-associated TF, is activated in inflammatory and autoimmune responses and is up-regulated at the site of AS lesions. HSF-1 reduces bile acid sequestration and increases circulating lipoproteins by up-regulating Cholesterol 7α-hydroxylase/Multidrug transporter 1. The deletion of HSF-1 inhibits heat shock and stress responses and decreases the levels of plasma lipoproteins, thereby inhibiting AS progression (121).

**Figure 2:**
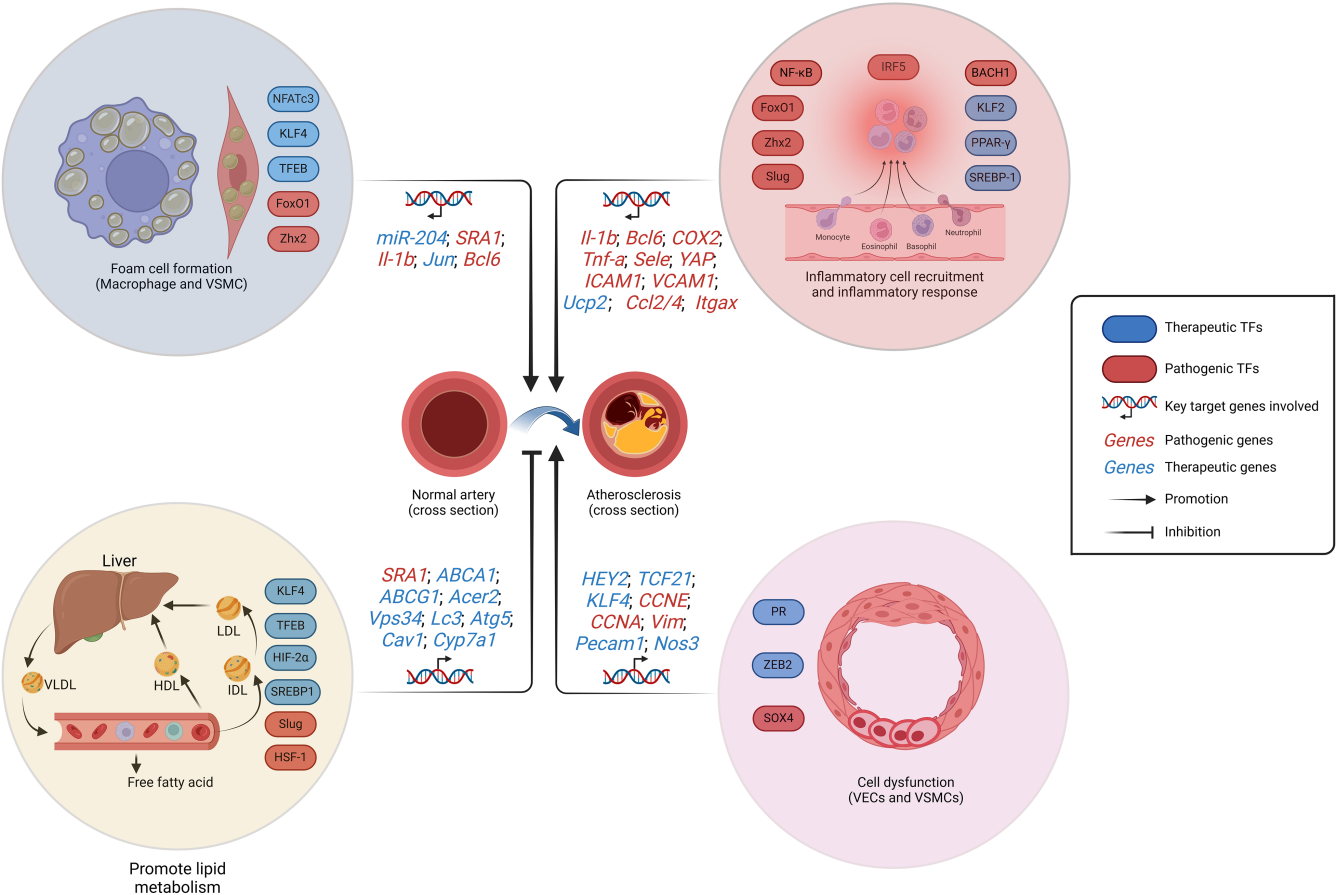
Schematic representation of the regulation of AS-related TFs. The formation of foam cells, cellular dysfunction, and the initiation of an inflammatory response contribute to the development of AS. While the promotion of lipid metabolism restrains AS progression. VSMCs and macrophages constitute the formation of foam cells. VSMCs dysfunction is manifested by pathological proliferation and the transformation into chondroblast. LDL, low-density lipoprotein; VLDL, very low-density lipoprotein; HDL, high-density lipoprotein; IDL, intermediate density lipoprotein; AS, atherosclerosis. Created with BioRender.com.

In the forthcoming years, TF-regulated clonal hematopoiesis may emerge as a focus in the investigation of AS. While the clonal hematopoiesis was found to be augmented in the elderly individuals and exhibited a strong correlation with AS (122). The pathogenesis of AS has been linked to a deficiency in clonal hematopoiesis-related TFs such as TET2 and DNMT3A due to the mutation of homologous genes, as reported (123, 124). Clonal hematopoiesis enhances the production and recruitment of inflammatory cells to the lesion, thereby exacerbating the inflammatory response, such as the IL-6-mediated response associated with macrophages (125). The elucidation of more mutation sites in TFs is imperative for a comprehensive understanding of the association between clonal hematopoiesis and AS in future studies. In short, whether it is inflammation response, SMC phenotypic switching, lipid metabolism or foam cell formation, TFs are involved in all stages of AS progression (Figure 2). Fortunately, there are currently quite a number of drugs involved in the regulation of these TFs in the treatment of AS.

## Pulmonary arterial hypertension-related TFs

Pulmonary arterial hypertension (PAH) is a kind of PH, which is localized to the pulmonary vasculature. Its common pathological features are vasoconstriction, inflammatory infiltration, and thrombosis caused by vasoactive substances secreted by immune cells in the circulating blood, cytokines, and platelet activation (126). Endothelial dysfunction caused by metabolic shifts in the intima of pulmonary arterial endothelial cells (PAECs) in response to metabolic shifts, tissue factors, and other factors, causes further loss of endothelial integrity and the formation of *in situ* thrombus; and the loss of vascular integrity and obstruction caused by intimal PASMCs in the tunica media cause vasoconstriction and vascular obstruction due to metabolic shifts and loss of calcium homeostasis. Furthermore, pulmonary artery fibroblasts (PAFs) in the adventitia grow out of control in response to different factors, causing vascular fibrosis and vascular stiffness (127, 128). A continuous increase in pulmonary vascular resistance increases the susceptibility of the patient to life-threatening right heart failure.

PAECs regulate multiple vascular functions, including vasodilation and vasoconstriction, growth and migration of PASMCs, regulation of thrombus formation, and inflammatory responses (126). Numerous TFs are involved in regulating these functions. FoxM1, another member of the Fox family, regulates the cell cycle. Dai et al. reported that FoxM1 is significantly up-regulated in the lung tissues of patients with idiopathic pulmonary arterial hypertension (IPAH) and PH mice. They revealed a mechanism by which the PAECs present in PH are interoperable with PASMCs. In the IPAH model, researchers detected elevated expression of endothelial dysfunction-associated factors, most notably CXCL12, inducing elevated expression of FoxM1 and promoting the proliferation of PASMCs (10). The present study illustrated the mechanism of EC-SMC crosstalk facilitated by FoxM1; however, it remains insufficient to comprehend the initiation of PAH. Further investigations are expected to elucidate the association between endothelial dysfunction, PASMCs proliferation, and vasoconstriction. In addition to AS, KLF4 functions as a defender in PAH. Shatat et al. reported that KLF4 was downregulated in the lung tissues of patients with IPAH. In PAECs, KLF4 promoted the expression of eNOS, endothelin receptor type B, and Prostaglandin I2 Synthase for vasodilatation, antiproliferative, antiplatelet attachment, and antithrombotic effects, whereas KLF4 inhibited the expression of EDN1 to reduce the vasoconstrictive effect and pro-proliferative effect of ET-1 (35). The current understanding of KLF4 is limited to PAECs, and its effect on PASMCs and right ventricular cardiomyocytes still needs to be explored to comprehend the global progression of PAH. The absence of Sox17 in IA inhibits the proliferation of VECs, and thus Sox17 could be playing a pathogenic role in PAH. Interestingly, Park et al. reported that the lack of Sox17 in PAECs of PAH mice up-regulated hepatocyte growth factor/c-Met signaling and caused pathological remodeling of the pulmonary vasculature, manifested as the proliferation of PAECs, displaying predominantly in contrast to the phenomenon in IA. In addition, Notch signaling inhibited Sox17 expression in VECs (31). *BMPR2* mutations are one of the most important genetic features of patients with PH, and BMPR2 deficiency promotes apoptosis in PAECs (129, 130). Alastalo et al. demonstrated that the mechanism of BMP-mediated endothelial homeostasis is dependent on the PPAR-γ/β-catenin transcriptional complex and identified apelin as a target of this complex. Apelin enhances the survival, proliferation, and migration of PAECs to promote endothelial homeostasis. The paracrine effect of it, however, exhibited an inverse phenotype in PASMC (131). The thiazolidinediones currently in clinical use fail to meet the requirements for PAH treatment, necessitating a focus on developing drugs that exhibit greater compatibility with PPARγ ligands, such as nitroalkene derivatives of unsaturated fatty acids. Other factors contributing to endothelial dysfunction include the endothelial-to-hematopoietic transition (EHT). Liang et al. conducted lineage tracing and reported that endothelial-derived cells transformed into hematopoietic cells with myeloid features during the progression of PH in mice. Runx1 is one of the TFs involved in EHT; its inhibition *in vivo* suppresses EHT and prevents these transformed hematopoietic cells from exiting the bone marrow (132, 133). The distinction in EHT extents between PH with left heart diseases and only PAH needs further clinical studies since Runx1 is highly expressed in circulating endothelial progenitor cells, which contributes a more comprehensive understanding of the relationship between EHT and PH.

Impaired energy metabolism in PASMCs is associated with the development of PAH (134). Li et al. reported that ALDH1A3, a glycolysis and cell proliferation-related enzyme, was up-regulated in patients with PAH. ALDH1A3 up-regulates cell cycle and metabolism-related genes such as *CCNA2* and *PKM2* by targeting TF NFYA and H3K37ac in PASMCs, promoting the proliferation of PASMCs and aggravating small pulmonary artery obstruction (135). This targeting effect, however, exhibits an opposite impact on PAECs. Therefore, future clinical translational studies should consider PASMCs-sensitive inhibitors. Thickening of the tunica media and formation of neointima constitute important pathologic features of PAH (128). Steffes et al. conducted a single-cell analysis and demonstrated that Notch3-labeled PASMCs predominate in neointimal cells, suggesting that Notch signaling is required for the formation of neointima (136). However, the relationship between neointima formation and media thickness induced by Notch3 and the inflammatory response remains unknown; nevertheless, it involved in the progression of PAH. PASMCs-mediated vasoconstrictor effects directly increase vascular resistance and cause PAH (137). Barnes et al. reported that TF HIF-1α in PASMCs attenuated the vasoconstrictive capacity of PASMCs by restricting myosin light chain phosphorylation and improved vascular remodeling by activating p27 in a hypoxia-induced mouse model of PH (26). Kurosawa et al. conducted a high-throughput compound screening by inhibiting PASMC proliferation and selected cyclamidomycin for their study. In addition to antiproliferative effects, cyclamidomycin inhibited the expression of HIF-1α and NF-κB to promote aerobic metabolism and suppress inflammation. Moreover, cyclamidomycin elevated the expression of Nrf2 to reduce the level of OS (48).

Massive proliferation of PAFs in the adventitia causes the thickening and stiffening of vessel walls and loss of pulmonary artery elasticity-important pathologic features of PAH (138). Li et al. identified that TF Elk-1 in PAFs mediated JNK-induced 15- Lipoxygenase expression and regulated p27 expression and nuclear translocation in a hypoxia-induced PAH model, causing elevated cyclin A and CDK2 expression and accelerating the progression of G1-S and S cycles and promoting the proliferation of PAFs (139). However, whether Elk-1 is related to the sudden increase of α-SMA in the tunica adventitia myofibroblast and the molecular basis remain unclear, which will enrich our understanding of the tunica adventitia in PAH and comprehend the connection with the tunica media. Regarding the phenotypic transition of PAFs, Chen et al. demonstrated that the TGF-β1/Smad3 signaling in PAFs inhibited the 5-HT-induced transition of PAFs to myofibroblasts, suppressed the production of connective tissue growth factor and ECM, and reduced the migration ability of PAFs (140).

**Figure 3:**
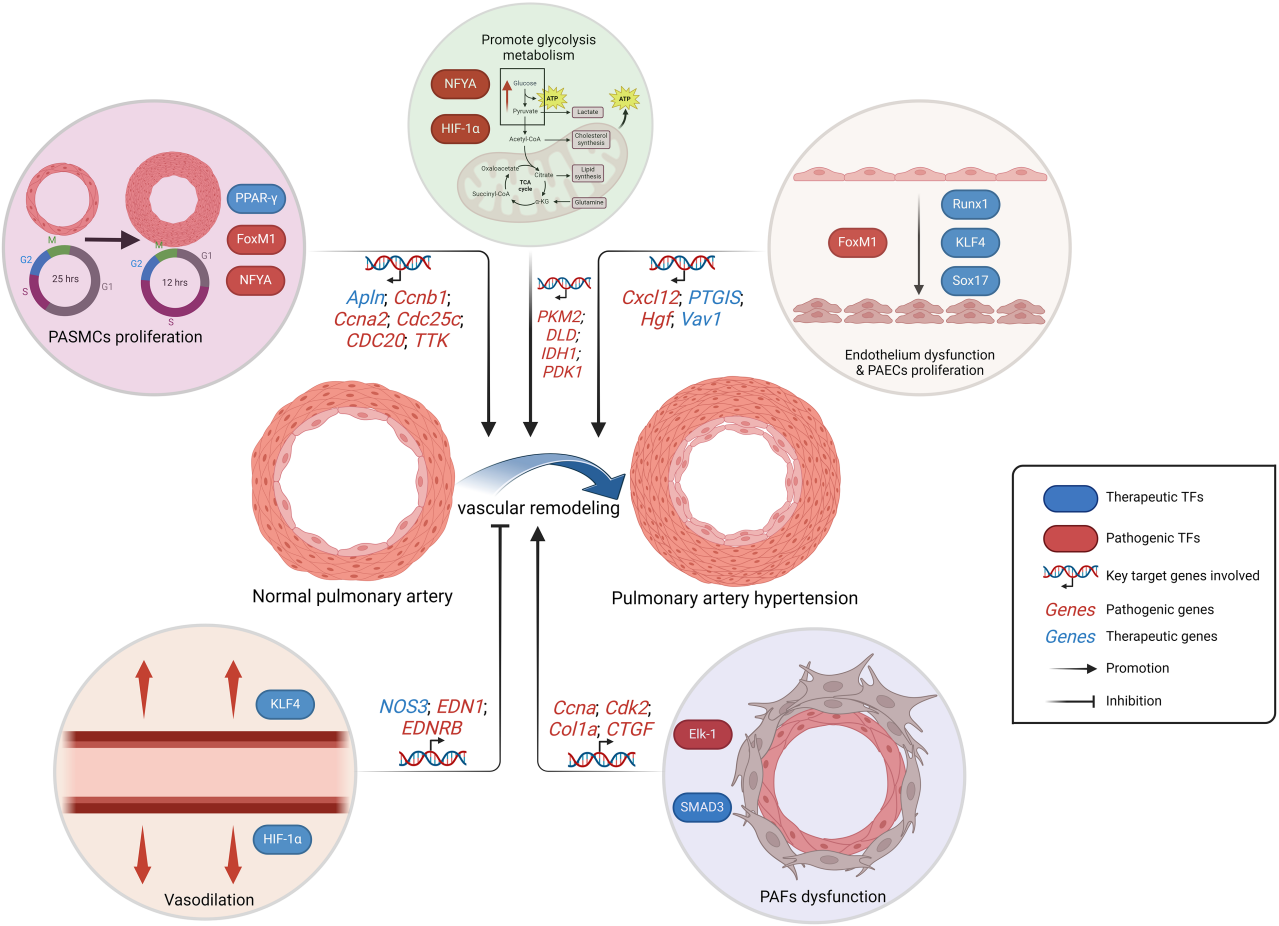
Schematic representation of the regulation of PAH-related TFs. The alteration in metabolic pattern, the excessive proliferation of PAECs and PASMCs, as well as the dysfunction of PAECs and PAFs, collectively contribute to pulmonary artery remodeling, which is responsible for the progression PAH. While the vasodilatory effect inhibits vascular remodeling. PAECs, pulmonary artery endothelial cells; PASMCs, pulmonary artery smooth muscle cells; PAFs, pulmonary artery fibroblasts. Created with BioRender.com.

In brief, TFs are substantially involved in the functional regulation of PAECs, PASMCs and PAFs, as well as in the regulation of a large number of intracellular signaling pathways. In addition, they are also indispensable in inflammation, oxidative stress, and metabolic pathways, and show great clinical translational potential (Figure 3). Currently, IPAH still constitutes a significant proportion, necessitating further exploration of genetic heterogeneity to enhance the comprehension of PAH (141). Consequently, TFs play a pivotal role in IPAH research. Future studies on PAH-related TFs will persist within the context of hypoxia-related vascular remodeling, which accompanied by severe cell proliferation and angiogenesis. The HIF TF family, as the mainly part in this background, has been extensively studied in PASMCs, PAECs and fibroblasts, exhibiting intricate cross talk with ECs proliferation and ROS production (142). Clinical translation targeting HIF family should be expedited in the future, since the drug such as 2- Methoxyestradiol, which targets both HIF-1α and HIF-2α, may potentially assume a prominent role in the treatment of PAH in the context of tumor-related studies (143, 144).

## Vasculitis-related TFs

Vasculitis refers to a group of diseases characterized by autoimmune disorders, infiltration of inflammatory cells in the vessel wall, perivascular tissues, and vessel lumens, thus causing vascular damage (145). Its major pathological changes include fibrin deposition, collagen fiber degeneration, necrosis of VECs and VSMCs, inflammatory cell infiltration such as neutrophils and lymphocytes, thrombosis, and altered blood composition. Its primary pathological classifications include ANCA-associated vasculitis (AAV), granulomatous vasculitis, allergic vasculitis, and segmental inflammation and necrosis (146). Patients with severe vasculitis who are not treated promptly could suffer life-threatening complications due to organ involvement and even organ failure such as aneurysm, heart failure, and kidney failure (147).

The two major areas to be investigated in vasculitis include the source of tissue-infiltrating inflammatory cells and the mechanisms of different kinds of tissue injury. Numerous studies on TFs have been conducted. For the large-vessel vasculitis, Takayasu arteritis is characterized by loss of immune tolerance, functional bias to Th1 and Th17, and mast cell-mediated vascular remodeling (145, 148, 149). Régnier et al. studied pro-inflammatory T-cell differentiation in TAK and reported that interferon-related gene expression was up-regulated in T-cells from patients with Takayasu disease by transcriptome analysis and that the JAK-STAT pathway was activated. The inhibition of this pathway significantly reduced T-cell activation, decreased polarization to Th1 and Th17, and increased the Treg ratio (150). The flow cytometry analysis of TAK patients compared to healthy donors in this study revealed a significant increase in p-STAT5 levels in both CD4^+^ and CD8^+^T cells, indicating its potential diagnostic value. AAV is identified as extravascular inflammation mediated by microvascular endothelial inflammation, leading to tissue injury, vascular fibrosis, and vascular dysfunction (151). When it comes to renal impairment, the activated and impaired VECs become a cause of necrotizing crescentic glomerulonephritis (152). Choi et al. reported elevated NF-κB expression in the glomeruli of AAV mouse models and patients, existing as a p50/p65 dimer. ANCA-stimulated neutrophils induced endothelial NF-κB expression in a cell contact-independent manner thus recruiting neutrophils to the endothelium. Subsequent activation of VECs is mediated partially by the release of TNF-α from ANCA-activated neutrophils (36). The activation of NF-kB exhibited a strong positive correlation with both the inflammatory response and the number of crescent glomeruli in mice, which reflect the severity of AAV, thereby indicating its potential prognostic significance. Kawasaki disease is marked by damage to coronary arteries and is the predominant vasculitic disease in children (153). This acute inflammatory response is primarily mediated by innate immune cells and accompanied by infiltration of IgA^+^ plasma cells and CD8^+^ T cells. Concurrently, there is an observed presence of proliferating myofibroblasts in the tunica intima (154). NFAT2 expression is up-regulated in Kawasaki disease (KD) and disrupts vascular endothelial cell homeostasis. The NFAT pathway is currently the only molecular target with clinical therapeutic relevance (155). Huang et al. demonstrated in human coronary artery endothelial cells that TF FOXO4 binds NFAT2 to down-regulate its expression, thereby increasing cadherin 5 levels to rescue the disruption of endothelial junctions by NFAT2 (156). The clinical investigations demonstrated a remarkable elevation in NFAT2 mRNA levels and firefly luciferase activity among KD patients, while the remaining four NFAT1 to NFAT5 are present at basal levels. This finding holds great potential as a valuable diagnostic marker. Giant cell arteritis (GCA) is another class of large-vessel vasculitis characterized by expression of NOTCH in T cells, and NOTCH is critical to Treg cell survival (157). The pathogenesis of GCA primarily involves the recruitment of T cells and monocytes mediated by activated dendritic cells, accompanied by the secretion of ROS and MMPs by multinucleated macrophages in tunica media, resulting in compromised vascular wall integrity (158). Jin et al. reported that CD8^+^ Treg cells from patients with GCA lacked anti-inflammatory function, whereas NOTCH1 and NOTCH4 signaling were up-regulated in CD4^+^T cells and CD8^+^ Treg cells, respectively. Meanwhile, these results serve as indications of the gravity of autoimmune vascular inflammation. Further studies have revealed that this defective NOTCH4 up-regulation in CD8^+^ Treg cells prevents NOX2 from translocating to the cell surface by vesicular transport and recycles NOX2 from the cell surface into the endosomes. In addition, high expression of NOTCH4 deprives CD8^+^ Treg cells of NOX2^+^ exosome secretion, failing to combat vascular inflammation (159). Sato et al. identified a population of CD4^+^T cells with high proliferative potential and stem cell-like features by single-cell transcriptome analysis. In arteritis lesions, the investigators reported a tertiary lymphoid structure in the perivascular adventitia, where CD4^+^T cells were localized and generated two effector cell populations, namely, EOMES^+^ cytotoxic T cells and BCL6^+^ follicular helper T cells, by expressing TF TCF1. Highly TCF1-expressing CD4^+^T cells proliferate and infiltrate *in situ*, produce effector T cells, and cause chronic and persistent GCA by expressing IL-7R (160). The CD4^+^ T cells exhibiting high levels of TCF1 expression undergo proliferation and differentiation, ultimately giving rise to pathological effector T cells that attack the vascular wall. This group of cells is recognized as the primary instigator of the disease. Uveitis is a typical clinical manifestation of leukemia, and Th17 and its related TFs are important factors in its pathogenesis (161). Yang et al. applied berberine to inhibit the activation of STAT3, which reduced the differentiation of CD4^+^T cells to Th17 cells and the production of IL-17, thereby suppressing the inflammatory response (162).

**Figure 4:**
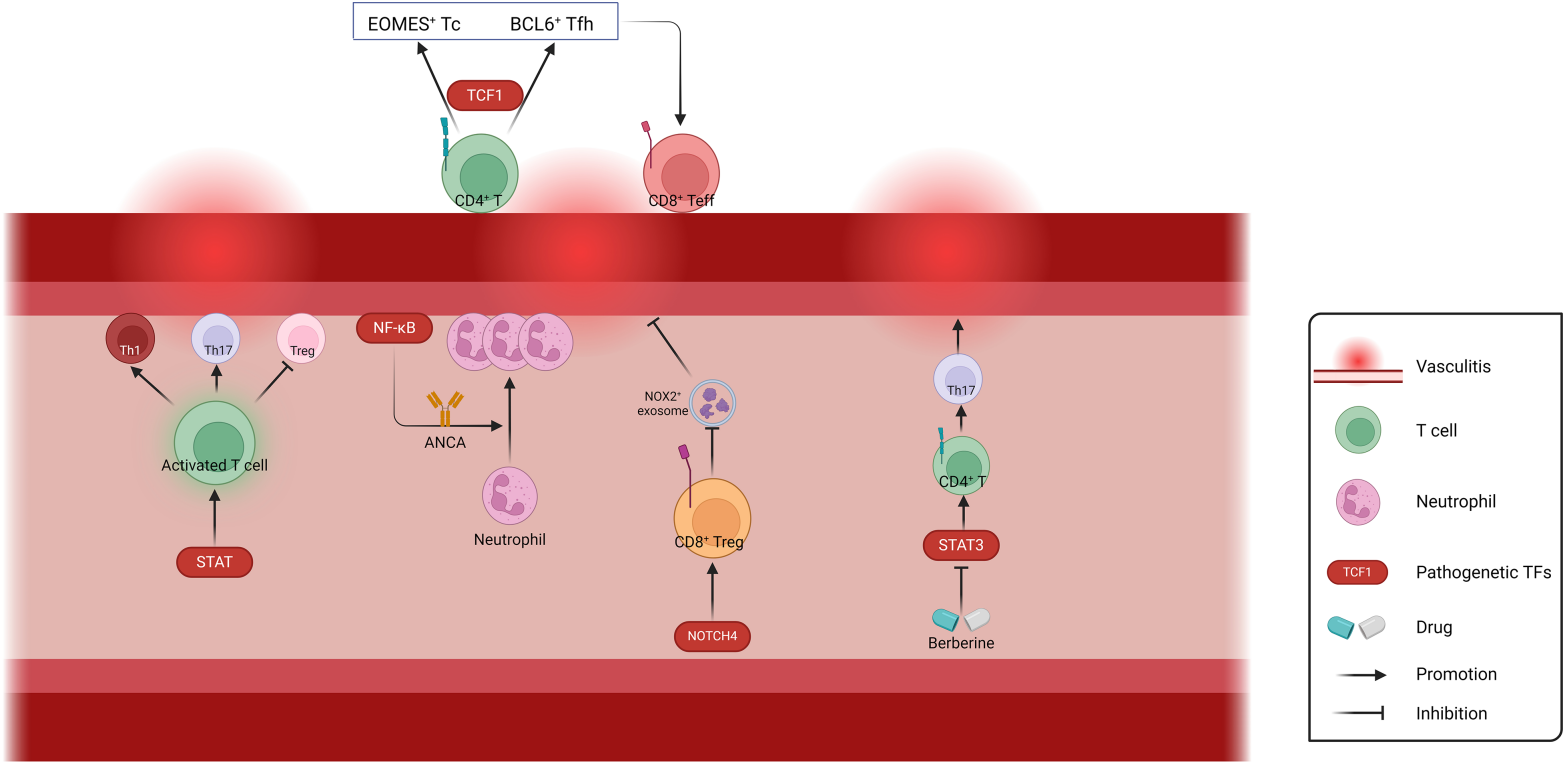
Schematic representation of the regulation of Vasculitis-related TFs. The involvement of TFs in T cell activation and differentiation, neutrophil activation and recruitment, as well as exosome-mediated biological processes, play crucial roles in the regulation of vascular wall inflammation. ANCA, anti-neutrophil cytoplasmic antibodies; Th, helper T cell; Tc, cytotoxic T cell; Tfh, follicular helper T cell; Teff, effector T cell; Treg, regulatory T cell. Created with BioRender.com.

In a word, current studies on vasculitis-related TFs mainly focus on the regulation of inflammatory responses related to T lymphocytes and neutrophils (Figure 4). Further studies are needed in other pathological types of vasculitis, such as autoimmune IgA vasculitis. When blood vessels undergo shear stress, activation of the mechanosensory ion channel molecule PIEZO1 is accompanied by the induction of innate immune response. Meanwhile, the absence of PIEZO1 also exhibits to ablate autoinflammation (163). It has been demonstrated that the TF NFAT/YAP complex induces PIEZO1 in the context of shear stress, which is worthy of investigation in autoimmune IgA vasculitis (13). With the increasing involvement of physics in vascular diseases research, inflammation related to physical factors such as shear stress may become a focal point in studying TFs involved in vasculitis.

## Summary and perspective

The epigenetic regulatory capacity of TFs is altered by pathogenic factors and influences disease progression. In addition, mutations in TF-DNA interactions function as key drivers of phenotypic variations and influence disease susceptibility (164). Therefore, it is essential to investigate the role of TFs in disease pathogenesis. TFs are implicated in the progression of vascular diseases by epigenetically regulating the proliferation, migration, differentiation, OS, inflammation, and metabolism of VECs and VSMCs. In the present study, we summarize the mechanisms by which TFs mediate the progression of vascular diseases via epigenetic regulation of ECs and SMCs, conclude the known and potential drug therapeutic targets, and propose diagnostic or therapeutic strategies to address the multiple mechanisms by which TFs regulate epigenetic transcriptional deregulation (Tables 1–4).

**Table 1:** Aneurysm-related TFs and potential drug targets.

TFs	Cell types	Targets	Effects	Drugs	References
VDR	VECs	MAPK signaling	Anti-inflammatory; anti-angiogenesis	Calcitriol	(84)
TFEB	VSMCs	Bcl2	Anti-apoptosis	Cyclodextrin	(58)
KLF15	VSMCs	p53-p300	Cope with stress correctly	Curcumin	(19)
ZEB2	VECs	CSE/H_2_S	Suppress ER stress	Entinostat	(69)
STAT1	Macrophages	STAT1-HIF1α	Inhibit M1 polarization	Bemcentinib	(90)
NRF2	Macrophages	NLRP3 signaling	Anti-inflammatory; Anti-apoptosis;	Oltipraz	(165)
NF-κB	VSMCs	TLR4 signaling	Anti-inflammatory; suppress ECM degradation	Taxifolin	(166)
STAT3	Macrophages	MMP9	Prevent elastin degradation	Imatinib	(167)
PPARγ	VSMCs	NFAT-NF-κB	Suppress SMC phenotypic modulation, proliferation and migration	Pioglitazone	(168)

VECs, vascular endothelial cells; MAPK, mitogen-activated protein kinase; VSMCs, vascular smooth muscle cells; CSE/H_2_S, cystathionine γ-lyase/hydrogen sulfide; ER, endoplasmic reticulum; STAT, signal transducers and activators of transcription; HIF, hypoxia-inducing factor; NLRP3, NOD-, LRR- and pyrin domain-containing 3; TLR4, toll-like receptor 4; MMP9, matrix metalloproteinase 9; NFAT, nuclear factor of activated T cells; NF-κB, nuclear factor kappa-B.

**Table 2:** AS-related TFs and potential drug targets.

TFs	Cell types	Targets	Effects	Drugs	References
KLF4	VSMCs; VECs	SRA	Inhibit foam cell formation	Formononetin	(101)
PR	VSMCs	CCNA; CCNE	Antiproliferative	Progesterone	(110)
PPARγ	VSMCs	MCP-1	Limit monocyte chemoattractant	Pioglitazone	(113)
SREBP-1	VSMCs	Caveolin-1	Anti-inflammatory; improve lipid metabolism	Curcumin	(117)
BACH-1	VECs	YAP	Anti-inflammatory	Rosuvastatin	(14)
KLF2	VECs	UCP2	Anti-inflammatory	Resveratrol	(112)
HIF-2α	Adipocyte	ACER2	Accelerate cholesterol clearance	Roxadustat	(49)
SOX4	VECs	Mesenchymal markers	Anti-EndoMT effect	Metformin	(30)
STAT3	Macrophages	SH2 domain	Anti-inflammatory	Luteolin	(169)
KLF14	Hepatocyte	ApoA-I	Increase HDL-C level and cholesterol efflux rate	Perhexiline	(170)
ER	VSMCs	CSE/H_2_S	Decrease plasma cholesterol level	Estrogen	(171)
TFEB	Macrophages	LC3; p62	Promote the autophagy-lysosomal system	Trehalose	(172)
NFAT	PBMCs;VECs	Pro-inflammatory genes	Suppress plaque inflammation	A-285222	(173)
AP-1	VECs	CD137	Anti-inflammatory	Phthalides	(174)
STAT1	Macrophages	AMPK-STAT1-STING signaling	Anti-inflammatory; improve lipid metabolism	Balasubramide derivative 3C	(175)
NF-κB	VECs	Pro-inflammatory genes	Anti-inflammatory; ameliorate endothelial dysfunction	Apabetalone	(176)
SP-1	Macrophages	PSRC1	Anti-inflammatory	Mithramycin	(177)
STAT6	Macrophages	PI3K/Akt-NF-κB signaling	Promote M2 activation	Protocatechuic acid	(178)
NRF2	VECs	HO-1	Anti-inflammatory; anti-oxidation	Cardamonin	(179)

SRA, scavenger receptor A1; CCNA/CCNE, cyclinA/E; MCP-1, monocyte chemoattractant protein-1; YAP, yes-associated protein; UCP2, uncoupling protein 2; ACER2, alkaline ceramidase 2; EndoMT, endothelial-to-mesenchymal transition; SH2, src homology domain 2; PBMCs, peripheral blood mononuclear cells; AMPK, adenosine 5′-monophosphate (AMP)-activated protein kinase; STING, stimulator of interferon genes; PSRC1, proline/serine-rich coiled-coil protein 1; PI3K/Akt, phosphatidylinositol-3-kinase/Akt; M2, type 2 polarized macrophages; HO-1, heme oxygenase-1.

**Table 3:** PAH-related TFs and potential drug targets.

TFs	Cell types	Targets	Effects	Drugs	References
FOXM1	PASMCs	CCNB1; CCNA2	Antiproliferative	Thiostrepton	(10)
SOX17	PAECs	HGF/c-Met signaling	Antiproliferative	Crizotinib	(31)
Notch3	PASMCs	Notch signaling	Suppress neointima formation	Dibenzazepine	(136)
HIF-1α	PASMCs	NF-κB	Antiproliferative	Celastramycin	(48)
HIF-2α	PAECs	CXCL12; ET-1	Antifibrosis	C76	(180)
STAT3; NFAT	Macrophages	SDF-1; MCP-1	Anti-inflammatory; anti-oxidation	Molecular hydrogen	(181)
ER	PASMCs	β-adrenergic receptor signaling	Suppress PASMCs hypertrophy	Genistein	(182)
NRF2	PASMCs	HO-1	Anti-inflammatory; anti-oxidation	Oxymatrine	(183)
RUNX2	PASMCs	Hippo signaling	Suppress arterial calcification	Magnesium sulfate	(184)

PASMCs, pulmonary artery smooth muscle cells; PAECs, pulmonary artery endothelial cells; HGF/c-Met, hepatocyte growth factor/c-mesenchymal-epithelial transition; CXCL12, chemokine (C-X-C motif) ligand 12; ET-1, endothelin-1; SDF-1, stromal cell-derived factor-1.

**Table 4:** Vasculitis-related TFs and potential drug targets.

TFs	Cell types	Targets	Effects	Drugs	References
STATs	T lymphocytes	JAK/STAT signaling	Suppress Th1/Th17 polarization	Ruxolitinib	(150)
Notch-1	CD4^+^T cells	mTORC1	Suppress Th1/Th17 polarization	DAPT	(185)
STAT6	M2 macrophages	Pro-fibrotic genes; M2 marker genes	Anti-inflammatory; antifibrosis	Leflunomide	(186)
STAT3	CD4^+^T cells	Differentiation-related genes	Suppress Th17 polarization	Berberine	(162)
NF-κB	PAECs	AMPK/mTOR/NF-κB signaling	Anti-inflammatory; anti-apoptosis	Liraglutide	(187)
IRF3	VECs	Pro-inflammatory genes	Suppress DNA-induced inflammation	LL37	(188)

JAK, janus kinase; mTORC1, mammalian target of rapamycin complex 1.

Tissue-specific TFs are known to selectively regulate gene expression. The DNA binding of target genes is specific. Each TF regulates only one set of gene targets, such that targeting aberrantly expressed TFs could be an effective strategy to treat diseases. However, except for nuclear hormone receptors such as PR, which can be regulated by ligand-binding domains, a few small molecule drugs can directly target TFs, which is partially attributed to the structural disorder of TFs and the lack of small molecule-binding pockets (15). Small molecule inhibitors targeting interactions of TFs with other proteins, molecular gels, and monomer degraders can modulate the stability of TFs, or Proteolysis-targeting chimeras degradation techniques targeting TFs can be used for drug design (189). However, almost all therapeutic strategies for TFs mentioned in the present study are limited by the current technology that places TFs in their signaling pathways and modulates them with existing drugs or small molecule compounds to treat the disease. The immediate application of TF therapy lies in its potential to replace coding gene defects, such as those associated with aortic aneurysm rupture caused by *SMAD3* mutations (7). The challenge lies in the complexity of comprehending the extent to which target cells can effectively accept and regulate the expression of target genes. In addition to its application in cancer therapy, chimeric antigen receptor is also anticipated to find utility in vasculitis or other inflammatory vascular diseases. The modification of T cells enables TFs to exert an immune regulatory function within the inflammatory microenvironment or alter the differentiation trajectory of activated T cells for enhanced control over the inflammatory response. TFs constitute an important link in the development of cell engineering, and understanding the alterations of TFs on cell progression contributes to the formulation of therapeutic strategies. Joung et al. applied single-cell transcriptome and chromatin accessibility analyses to construct a targeted differentiated TFs atlas, mapped the effects of TFs overexpression to reference cell types with single-cell resolution, and established a cellular disease model by sequencing a screening library of targeted TFs, including SMCs, ECs, and EMT SMCs, that are intricately related to vascular diseases. In addition, the researchers conducted mimetic time-series analysis to screen and validate that over a quarter of TFs overexpressed affected the differentiation of human embryonic stem cells and mapped the regulatory network of TFs through ATAC data to develop a transcription factor screen for predicting reference cell types (190).

Currently, TFs-related research methods include electrophoretic mobility shift assay, luciferase reporter assay, yeast one-hybrid assay, and CHIP. Researchers have been applying TRANSFAC and JASPAR databases to systematically recognize the structure and function of TFs and predict their potential regulatory functions and signaling pathway integration (191, 192). However, the understanding of TFs is still limited. For example, although the DNA-binding domains of TFs are known and can be used to study disease pathogenesis, the transcriptional effector domains, which are also important components of TFs, are relatively poorly understood. Researchers have already conducted large-scale effector activity assays of TFs and annotated thousands of effector domains; however, the annotation of about 60% of transcriptional effector domains needs improvement. Therefore, high-throughput TFs effector domain analysis in vascular-related diseases could emerge as a significant research direction in the future (193, 194). Meanwhile, more comprehensive pseudotime analysis ought to be employed in future studies to examine the trajectories of pluripotent stem/progenitor cells, aiming to unveil the occurrence of cell fate switching. Vascular diseases encompass a multitude of cellular functional transformations, including the transition of VECs into hematopoietic cells, mesenchymal cells, or chondroblasts, as well as senescence-associated secretion phenotypes exhibited by VSMCs. Recent investigations on the epigenetic regulation governing the destiny of embryonic epidermal stem cells mediated by the TF SOX9 hold instructive implications for subsequent research endeavors in vascular diseases (195). The integration of the previously proposed transcriptional factor activity sequencing technology will enable a more comprehensive analysis of cell trajectory in the regulation of vascular cell fate (196).

## References

[1] Ptashne M Gann AA. Activators and targets. Nature. (1990) 346(6282):329–31. 10.1038/346329a0 2142753

[2] Lambert SA Jolma A Campitelli LF Das PK Yin Y Albu M The human transcription factors. Cell. (2018) 172(4):650–65. 10.1016/j.cell.2018.01.029 29425488 PMC12908702

[3] Sainsbury S Bernecky C Cramer P. Structural basis of transcription initiation by RNA polymerase II. Nat Rev Mol Cell Biol. (2015) 16(3):129–43. 10.1038/nrm3952 25693126

[4] Maniatis T Goodbourn S Fischer JA. Regulation of inducible and tissue-specific gene expression. Science. (1987) 236(4806):1237–45. 10.1126/science.3296191 3296191

[5] Quintana RA Taylor WR. Cellular mechanisms of aortic aneurysm formation. Circ Res. (2019) 124(4):607–18. 10.1161/CIRCRESAHA.118.313187 30763207 PMC6383789

[6] Sakalihasan N Limet R Defawe OD. Abdominal aortic aneurysm. Lancet Lond Engl. (2005) 365(9470):1577–89. 10.1016/S0140-6736(05)66459-8 15866312

[7] Ye P Chen W Wu J Huang X Li J Wang S GM-CSF contributes to aortic aneurysms resulting from SMAD3 deficiency. J Clin Invest. (2013) 123(5):2317–31. 10.1172/JCI67356 23585475 PMC3635740

[8] Vaquerizas JM Kummerfeld SK Teichmann SA Luscombe NM. A census of human transcription factors: function, expression and evolution. Nat Rev Genet. (2009) 10(4):252–63. 10.1038/nrg2538 19274049

[9] Hannenhalli S Kaestner KH. The evolution of fox genes and their role in development and disease. Nat Rev Genet. (2009) 10(4):233–40. 10.1038/nrg2523 19274050 PMC2733165

[10] Dai Z Zhu MM Peng Y Jin H Machireddy N Qian Z Endothelial and smooth muscle cell interaction via FoxM1 signaling mediates vascular remodeling and pulmonary hypertension. Am J Respir Crit Care Med. (2018) 198(6):788–802. 10.1164/rccm.201709-1835OC 29664678 PMC6222462

[11] Zhang X Evans TD Chen S Sergin I Stitham J Jeong SJ Loss of macrophage mTORC2 drives atherosclerosis via FoxO1 and IL-1β signaling. Circ Res. (2023) 133(3):200–19. 10.1161/CIRCRESAHA.122.321542 37350264 PMC10527041

[12] Souilhol C Serbanovic-Canic J Fragiadaki M Chico TJ Ridger V Roddie H Endothelial responses to shear stress in atherosclerosis: a novel role for developmental genes. Nat Rev Cardiol. (2020) 17(1):52–63. 10.1038/s41569-019-0239-5 31366922

[13] Zhou T Gao B Fan Y Liu Y Feng S Cong Q Piezo1/2 mediate mechanotransduction essential for bone formation through concerted activation of NFAT-YAP1-ß-catenin. eLife. (2020) 9:e52779. 10.7554/eLife.52779 32186512 PMC7112954

[14] Jia M Li Q Guo J Shi W Zhu L Huang Y Deletion of BACH1 attenuates atherosclerosis by reducing endothelial inflammation. Circ Res. (2022) 130(7):1038–55. 10.1161/CIRCRESAHA.121.319540 35196865

[15] Bushweller JH. Targeting transcription factors in cancer—from undruggable to reality. Nat Rev Cancer. (2019) 19(11):611–24. 10.1038/s41568-019-0196-7 31511663 PMC8820243

[16] Pugsley MK Tabrizchi R. The vascular system. An overview of structure and function. J Pharmacol Toxicol Methods. (2000) 44(2):333–40. 10.1016/S1056-8719(00)00125-8 11325577

[17] Frismantiene A Philippova M Erne P Resink TJ. Smooth muscle cell-driven vascular diseases and molecular mechanisms of VSMC plasticity. Cell Signal. (2018) 52:48–64. 10.1016/j.cellsig.2018.08.019 30172025

[18] Levine AJ. P53, the cellular gatekeeper for growth and division. Cell. (1997) 88(3):323–31. 10.1016/S0092-8674(00)81871-1 9039259

[19] Haldar SM Lu Y Jeyaraj D Kawanami D Cui Y Eapen SJ Klf15 deficiency is a molecular link between heart failure and aortic aneurysm formation. Sci Transl Med. (2010) 2(26):26ra26. 10.1126/scitranslmed.3000502 20375365 PMC3003709

[20] Gomez D Kessler K Michel JB Vranckx R. Modifications of chromatin dynamics control Smad2 pathway activation in aneurysmal smooth muscle cells. Circ Res. (2013) 113(7):881–90. 10.1161/CIRCRESAHA.113.301989 23825360

[21] Ihling C Szombathy T Nampoothiri K Haendeler J Beyersdorf F Uhl M Cystic medial degeneration of the aorta is associated with p53 accumulation, bax upregulation, apoptotic cell death, and cell proliferation. Heart Br Card Soc. (1999) 82(3):286–93. 10.1136/hrt.82.3.286 PMC1729171 10455077

[22] Wan S George SJ Nicklin SA Yim APC Baker AH. Overexpression of p53 increases lumen size and blocks neointima formation in porcine interposition vein grafts. Mol Ther J Am Soc Gene Ther. (2004) 9(5):689–98. 10.1016/j.ymthe.2004.02.005 15120330

[23] Choudhury AR Ju Z Djojosubroto MW Schienke A Lechel A Schaetzlein S Cdkn1a deletion improves stem cell function and lifespan of mice with dysfunctional telomeres without accelerating cancer formation. Nat Genet. (2007) 39(1):99–105. 10.1038/ng1937 17143283

[24] Shi J Yang Y Cheng A Xu G He F. Metabolism of vascular smooth muscle cells in vascular diseases. Am J Physiol Heart Circ Physiol. (2020) 319(3):H613–31. 10.1152/ajpheart.00220.2020 32762559

[25] Butler TM Siegman MJ. High-energy phosphate metabolism in vascular smooth muscle. Annu Rev Physiol. (1985) 47:629–43. 10.1146/annurev.ph.47.030185.003213 3158271

[26] Barnes EA Ito R Che X Alvira CM Cornfield DN. Loss of prolyl hydroxylase 1 and 2 in SM22α-expressing cells prevents hypoxia-induced pulmonary hypertension. Am J Physiol Lung Cell Mol Physiol. (2023) 325(6):L741–55. 10.1152/ajplung.00428.2022 37847687 PMC11068430

[27] Owens GK Kumar MS Wamhoff BR. Molecular regulation of vascular smooth muscle cell differentiation in development and disease. Physiol Rev. (2004) 84(3):767–801. 10.1152/physrev.00041.2003 15269336

[28] Trimm E Red-Horse K. Vascular endothelial cell development and diversity. Nat Rev Cardiol. (2023) 20(3):197–210. 10.1038/s41569-022-00770-1 36198871 PMC9533272

[29] Sarkar A Hochedlinger K. The sox family of transcription factors: versatile regulators of stem and progenitor cell fate. Cell Stem Cell. (2013) 12(1):15–30. 10.1016/j.stem.2012.12.007 23290134 PMC3608206

[30] Cheng CK Lin X Pu Y Tse JKY Wang Y Zhang CL SOX4 Is a novel phenotypic regulator of endothelial cells in atherosclerosis revealed by single-cell analysis. J Adv Res. (2023) 43:187–203. 10.1016/j.jare.2022.02.017 36585108 PMC9811326

[31] Park CS Kim SH Yang HY Kim JH Schermuly RT Cho YS Sox17 deficiency promotes pulmonary arterial hypertension via HGF/c-met signaling. Circ Res. (2022) 131(10):792–806. 10.1161/CIRCRESAHA.122.320845 36205124 PMC9612711

[32] Hagedorn EJ Perlin JR Freeman RJ Wattrus SJ Han T Mao C Transcription factor induction of vascular blood stem cell niches in vivo. Dev Cell. (2023) 58(12):1037–51.e4. 10.1016/j.devcel.2023.04.007 37119815 PMC10330626

[33] Kunisaki Y Bruns I Scheiermann C Ahmed J Pinho S Zhang D Arteriolar niches maintain haematopoietic stem cell quiescence. Nature. (2013) 502(7473):637–43. 10.1038/nature12612 24107994 PMC3821873

[34] Rader DJ Daugherty A. Translating molecular discoveries into new therapies for atherosclerosis. Nature. (2008) 451(7181):904–13. 10.1038/nature06796 18288179

[35] Shatat MA Tian H Zhang R Tandon G Hale A Fritz JS Endothelial krüppel-like factor 4 modulates pulmonary arterial hypertension. Am J Respir Cell Mol Biol. (2014) 50(3):647–53. 10.1165/rcmb.2013-0135OC 24156273 PMC4068930

[36] Choi M Schreiber A Eulenberg-Gustavus C Scheidereit C Kamps J Kettritz R. Endothelial NF-κB blockade abrogates ANCA-induced GN. J Am Soc Nephrol JASN. (2017) 28(11):3191–204. 10.1681/ASN.2016060690 28687535 PMC5661273

[37] Fan Y Lu H Liang W Hu W Zhang J Chen YE. Krüppel-like factors and vascular wall homeostasis. J Mol Cell Biol. (2017) 9(5):352–63. 10.1093/jmcb/mjx037 28992202 PMC5907833

[38] Wu C Huang RT Kuo CH Kumar S Kim CW Lin YC Mechanosensitive PPAP2B regulates endothelial responses to atherorelevant hemodynamic forces. Circ Res. (2015) 117(4):e41–53. 10.1161/CIRCRESAHA.117.306457 26034042 PMC4522239

[39] SenBanerjee S Lin Z Atkins GB Greif DM Rao RM Kumar A KLF2 is a novel transcriptional regulator of endothelial proinflammatory activation. J Exp Med. (2004) 199(10):1305–15. 10.1084/jem.20031132 15136591 PMC2211816

[40] Lin Z Kumar A SenBanerjee S Staniszewski K Parmar K Vaughan DE Kruppel-like factor 2 (KLF2) regulates endothelial thrombotic function. Circ Res. (2005) 96(5):e48–57. 10.1161/01.RES.0000159707.05637.a1 15718498

[41] Bhattacharya R Senbanerjee S Lin Z Mir S Hamik A Wang P Inhibition of vascular permeability factor/vascular endothelial growth factor-mediated angiogenesis by the kruppel-like factor KLF2. J Biol Chem. (2005) 280(32):28848–51. 10.1074/jbc.C500200200 15980434

[42] Hoesel B Schmid JA. The complexity of NF-κB signaling in inflammation and cancer. Mol Cancer. (2013) 12:86. 10.1186/1476-4598-12-86 23915189 PMC3750319

[43] Soh UJK Dores MR Chen B Trejo J. Signal transduction by protease-activated receptors. Br J Pharmacol. (2010) 160(2):191–203. 10.1111/j.1476-5381.2010.00705.x 20423334 PMC2874842

[44] Baud V Karin M. Signal transduction by tumor necrosis factor and its relatives. Trends Cell Biol. (2001) 11(9):372–7. 10.1016/S0962-8924(01)02064-5 11514191

[45] Eelen G de Zeeuw P Simons M Carmeliet P. Endothelial cell metabolism in normal and diseased vasculature. Circ Res. (2015) 116(7):1231–44. 10.1161/CIRCRESAHA.116.302855 25814684 PMC4380230

[46] Lee JW Bae SH Jeong JW Kim SH Kim KW. Hypoxia-inducible factor (HIF-1)alpha: its protein stability and biological functions. Exp Mol Med. (2004) 36(1):1–12. 10.1038/emm.2004.1 15031665

[47] Chen C Pore N Behrooz A Ismail-Beigi F Maity A. Regulation of glut1 mRNA by hypoxia-inducible factor-1. Interaction between H-ras and hypoxia. J Biol Chem. (2001) 276(12):9519–25. 10.1074/jbc.M010144200 11120745

[48] Kurosawa R Satoh K Kikuchi N Kikuchi H Saigusa D Al-Mamun ME Identification of celastramycin as a novel therapeutic agent for pulmonary arterial hypertension. Circ Res. (2019) 125(3):309–27. 10.1161/CIRCRESAHA.119.315229 31195886

[49] Zhang X Zhang Y Wang P Zhang SY Dong Y Zeng G Adipocyte hypoxia-inducible factor 2α suppresses atherosclerosis by promoting adipose ceramide catabolism. Cell Metab. (2019) 30(5):937–51.e5. 10.1016/j.cmet.2019.09.016 31668872

[50] Baxter BT McGee GS Shively VP Drummond IA Dixit SN Yamauchi M Elastin content, cross-links, and mRNA in normal and aneurysmal human aorta. J Vasc Surg. (1992) 16(2):192–200. 10.1016/0741-5214(92)90107-J 1495142

[51] Dobrin PB Mrkvicka R. Failure of elastin or collagen as possible critical connective tissue alterations underlying aneurysmal dilatation. Cardiovasc Surg Lond Engl. (1994) 2(4):484–8. 10.1177/096721099400200412 7953454

[52] Huffman MD Curci JA Moore G Kerns DB Starcher BC Thompson RW. Functional importance of connective tissue repair during the development of experimental abdominal aortic aneurysms. Surgery. (2000) 128(3):429–38. 10.1067/msy.2000.107379 10965315

[53] López-Candales A Holmes DR Liao S Scott MJ Wickline SA Thompson RW. Decreased vascular smooth muscle cell density in medial degeneration of human abdominal aortic aneurysms. Am J Pathol. (1997) 150(3):993–1007. 9060837 PMC1857880

[54] Shah PK. Inflammation, metalloproteinases, and increased proteolysis: an emerging pathophysiological paradigm in aortic aneurysm. Circulation. (1997) 96(7):2115–7. 10.1161/01.CIR.96.7.2115 9337176

[55] Vorp DA Lee PC Wang DH Makaroun MS Nemoto EM Ogawa S Association of intraluminal thrombus in abdominal aortic aneurysm with local hypoxia and wall weakening. J Vasc Surg. (2001) 34(2):291–9. 10.1067/mva.2001.114813 11496282

[56] Johansson G Swedenborg J. Ruptured abdominal aortic aneurysms: a study of incidence and mortality. Br J Surg. (1986) 73(2):101–3. 10.1002/bjs.1800730205 3947894

[57] Salmon M Schaheen B Spinosa M Montgomery W Pope NH Davis JP ZFP148 (zinc-finger protein 148) binds cooperatively with NF-1 (neurofibromin 1) to inhibit smooth muscle marker gene expression during abdominal aortic aneurysm formation. Arterioscler Thromb Vasc Biol. (2019) 39(1):73–88. 10.1161/ATVBAHA.118.311136 30580567 PMC6422047

[58] Lu H Sun J Liang W Chang Z Rom O Zhao Y Cyclodextrin prevents abdominal aortic aneurysm via activation of vascular smooth muscle cell transcription factor EB. Circulation. (2020) 142(5):483–98. 10.1161/CIRCULATIONAHA.119.044803 32354235 PMC7606768

[59] Tanaka Y Yamada Y Ishitsuka Y Matsuo M Shiraishi K Wada K Efficacy of 2-hydroxypropyl-β-cyclodextrin in niemann-pick disease type C model mice and its pharmacokinetic analysis in a patient with the disease. Biol Pharm Bull. (2015) 38(6):844–51. 10.1248/bpb.b14-00726 26027824

[60] Gao P Zhang H Zhang Q Fang X Wu H Wang M Caloric restriction exacerbates angiotensin II-induced abdominal aortic aneurysm in the absence of p53. Hypertens Dallas Tex 1979. (2019) 73(3):547–60. 10.1161/HYPERTENSIONAHA.118.12086 30686087

[61] Gong J Zhou D Jiang L Qiu P Milewicz DM Chen YE In vitro lineage-specific differentiation of vascular smooth muscle cells in response to SMAD3 deficiency: implications for SMAD3-related thoracic aortic aneurysm. Arterioscler Thromb Vasc Biol. (2020) 40(7):1651–63. 10.1161/ATVBAHA.120.313033 32404006 PMC7316596

[62] Lu H Du W Ren L Hamblin MH Becker RC Chen YE Vascular smooth muscle cells in aortic aneurysm: from genetics to mechanisms. J Am Heart Assoc. (2021) 10(24):e023601. 10.1161/JAHA.121.023601 34796717 PMC9075263

[63] Chakraborty A Li Y Zhang C Li Y Rebello KR Li S Epigenetic induction of smooth muscle cell phenotypic alterations in aortic aneurysms and dissections. Circulation. (2023) 148(12):959–77. 10.1161/CIRCULATIONAHA.123.063332 37555319 PMC10529114

[64] Zhao G Fu Y Cai Z Yu F Gong Z Dai R Unspliced XBP1 confers VSMC homeostasis and prevents aortic aneurysm formation via FoxO4 interaction. Circ Res. (2017) 121(12):1331–45. 10.1161/CIRCRESAHA.117.311450 29089350

[65] Arif R Zaradzki M Remes A Seppelt P Kunze R Schröder H AP-1 Oligodeoxynucleotides reduce aortic elastolysis in a murine model of marfan syndrome. Mol Ther Nucleic Acids. (2017) 9:69–79. 10.1016/j.omtn.2017.08.014 29246325 PMC5608502

[66] Mikołajczyk K Spyt D Zielińska W Żuryń A Faisal I Qamar M The important role of endothelium and extracellular vesicles in the cellular mechanism of aortic aneurysm formation. Int J Mol Sci. (2021) 22(23):13157. 10.3390/ijms222313157 34884962 PMC8658239

[67] Sheinberg DL McCarthy DJ Elwardany O Bryant JP Luther E Chen SH Endothelial dysfunction in cerebral aneurysms. Neurosurg Focus. (2019) 47(1):E3. 10.3171/2019.4.FOCUS19221 31389675

[68] Beech DJ Kalli AC. Force sensing by piezo channels in cardiovascular health and disease. Arterioscler Thromb Vasc Biol. (2019) 39(11):2228–39. 10.1161/ATVBAHA.119.313348 31533470 PMC6818984

[69] Luo S Kong C Zhao S Tang X Wang Y Zhou X Endothelial HDAC1-ZEB2-NuRD complex drives aortic aneurysm and dissection through regulation of protein S-sulfhydration. Circulation. (2023) 147(18):1382–403. 10.1161/CIRCULATIONAHA.122.062743 36951067

[70] Lee S Kim IK Ahn JS Woo DC Kim ST Song S Deficiency of endothelium-specific transcription factor Sox17 induces intracranial aneurysm. Circulation. (2015) 131(11):995–1005. 10.1161/CIRCULATIONAHA.114.012568 25596186

[71] Incalza MA D’Oria R Natalicchio A Perrini S Laviola L Giorgino F. Oxidative stress and reactive oxygen species in endothelial dysfunction associated with cardiovascular and metabolic diseases. Vascul Pharmacol. (2018) 100:1–19. 10.1016/j.vph.2017.05.005 28579545

[72] Zhao W Yao M Zhang Y Xiong D Dai G Zhang J Endothelial cyclin I reduces vulnerability to angiotensin II-induced vascular remodeling and abdominal aortic aneurysm risk. Microvasc Res. (2022) 142:104348. 10.1016/j.mvr.2022.104348 35245516

[73] Aoki T Nishimura M Matsuoka T Yamamoto K Furuyashiki T Kataoka H PGE(2)-EP(2) signalling in endothelium is activated by haemodynamic stress and induces cerebral aneurysm through an amplifying loop via NF-κB. Br J Pharmacol. (2011) 163(6):1237–49. 10.1111/j.1476-5381.2011.01358.x 21426319 PMC3144537

[74] Hamann B Klimova A Klotz F Frank F Jänichen C Kapalla M Regulation of CD163 receptor in patients with abdominal aortic aneurysm and associations with antioxidant enzymes HO-1 and NQO1. Antioxid Basel Switz. (2023) 12(4):947. 10.3390/antiox12040947 PMC10135987 37107322

[75] De Martin R Hoeth M Hofer-Warbinek R Schmid JA. The transcription factor NF-kappa B and the regulation of vascular cell function. Arterioscler Thromb Vasc Biol. (2000) 20(11):E83–88. 10.1161/01.atv.20.11.e83 11073859

[76] Saito T Hasegawa Y Ishigaki Y Yamada T Gao J Imai J Importance of endothelial NF-κB signalling in vascular remodelling and aortic aneurysm formation. Cardiovasc Res. (2013) 97(1):106–14. 10.1093/cvr/cvs298 23015640

[77] Gimbrone MA García-Cardeña G. Endothelial cell dysfunction and the pathobiology of atherosclerosis. Circ Res. (2016) 118(4):620–36. 10.1161/CIRCRESAHA.115.306301 26892962 PMC4762052

[78] Zhao G Chang Z Zhao Y Guo Y Lu H Liang W KLF11 Protects against abdominal aortic aneurysm through inhibition of endothelial cell dysfunction. JCI Insight. (2021) 6(5):e141673. 10.1172/jci.insight.141673 33507881 PMC8021107

[79] Gross PL Aird WC. The endothelium and thrombosis. Semin Thromb Hemost. (2000) 26(5):463–78. 10.1055/s-2000-13202 11129402

[80] Shin IS Kim JM Kim KL Jang SY Jeon ES Choi SH Early growth response factor-1 is associated with intraluminal thrombus formation in human abdominal aortic aneurysm. J Am Coll Cardiol. (2009) 53(9):792–9. 10.1016/j.jacc.2008.10.055 19245972

[81] Peshkova IO Schaefer G Koltsova EK. Atherosclerosis and aortic aneurysm—is inflammation a common denominator? FEBS J. (2016) 283(9):1636–52. 10.1111/febs.13634 26700480

[82] Yuan Z Lu Y Wei J Wu J Yang J Cai Z. Abdominal aortic aneurysm: roles of inflammatory cells. Front Immunol. (2020) 11:609161. 10.3389/fimmu.2020.609161 33613530 PMC7886696

[83] Arthur JSC Ley SC. Mitogen-activated protein kinases in innate immunity. Nat Rev Immunol. (2013) 13(9):679–92. 10.1038/nri3495 23954936

[84] Martorell S Hueso L Gonzalez-Navarro H Collado A Sanz MJ Piqueras L. Vitamin D receptor activation reduces angiotensin-II-induced dissecting abdominal aortic aneurysm in apolipoprotein E-knockout mice. Arterioscler Thromb Vasc Biol. (2016) 36(8):1587–97. 10.1161/ATVBAHA.116.307530 27283745

[85] Muindi JR Peng Y Potter DM Hershberger PA Tauch JS Capozzoli MJ Pharmacokinetics of high-dose oral calcitriol: results from a phase 1 trial of calcitriol and paclitaxel. Clin Pharmacol Ther. (2002) 72(6):648–59. 10.1067/mcp.2002.129305 12496746

[86] Gao P Gao P Zhao J Shan S Luo W Slivano OJ MKL1 cooperates with p38MAPK to promote vascular senescence, inflammation, and abdominal aortic aneurysm. Redox Biol. (2021) 41:101903. 10.1016/j.redox.2021.101903 33667992 PMC7937568

[87] Lv BJ Li J Cheng X. T lymphocytes and aortic aneurysms. Sci China Life Sci. (2014) 57(8):795–801. 10.1007/s11427-014-4699-x 25104452

[88] Romain M Taleb S Dalloz M Ponnuswamy P Esposito B Pérez N Overexpression of SOCS3 in T lymphocytes leads to impaired interleukin-17 production and severe aortic aneurysm formation in mice–brief report. Arterioscler Thromb Vasc Biol. (2013) 33(3):581–4. 10.1161/ATVBAHA.112.300516 23329138

[89] Cheng Z Zhou YZ Wu Y Wu QY Liao XB Fu XM Diverse roles of macrophage polarization in aortic aneurysm: destruction and repair. J Transl Med. (2018) 16(1):354. 10.1186/s12967-018-1731-0 30545380 PMC6293547

[90] Han Y Li G Zhang Z Zhang X Zhao B Yang H. Axl promotes intracranial aneurysm rupture by regulating macrophage polarization toward M1 via STAT1/HIF-1α. Front Immunol. (2023) 14:1158758. 10.3389/fimmu.2023.1158758 37223093 PMC10200875

[91] Mosser DM Hamidzadeh K Goncalves R. Macrophages and the maintenance of homeostasis. Cell Mol Immunol. (2021) 18(3):579–87. 10.1038/s41423-020-00541-3 32934339 PMC7491045

[92] Salarian M Ghim M Toczek J Han J Weiss D Spronck B Homeostatic, non-canonical role of macrophage elastase in vascular integrity. Circ Res. (2023) 132(4):432–48. 10.1161/CIRCRESAHA.122.322096 36691905 PMC9930896

[93] Hu K Zhong L Lin W Zhao G Pu W Feng Z Pathogenesis-guided rational engineering of nanotherapies for the targeted treatment of abdominal aortic aneurysm by inhibiting neutrophilic inflammation. ACS Nano. (2024) 18(8):6650–72. 10.1021/acsnano.4c00120 38369729

[94] Khan MA Palaniyar N. Transcriptional firing helps to drive NETosis. Sci Rep. (2017) 7:1–16. 10.1038/srep41749 28176807 PMC5296899

[95] Libby P. The changing landscape of atherosclerosis. Nature. (2021) 592(7855):524–33. 10.1038/s41586-021-03392-8 33883728

[96] Chistiakov DA Melnichenko AA Myasoedova VA Grechko AV Orekhov AN. Mechanisms of foam cell formation in atherosclerosis. J Mol Med Berl Ger. (2017) 95(11):1153–65. 10.1007/s00109-017-1575-8 28785870

[97] Wang D Yang Y Lei Y Tzvetkov NT Liu X Yeung AWK Targeting foam cell formation in atherosclerosis: therapeutic potential of natural products. Pharmacol Rev. (2019) 71(4):596–670. 10.1124/pr.118.017178 31554644

[98] Yu XH Fu YC Zhang DW Yin K Tang CK. Foam cells in atherosclerosis. Clin Chim Acta Int J Clin Chem. (2013) 424:245–52. 10.1016/j.cca.2013.06.006 23782937

[99] Satterthwaite R Aswad S Sunga V Shidban H Bogaard T Asai P Incidence of new-onset hypercholesterolemia in renal transplant patients treated with FK506 or cyclosporine. Transplantation. (1998) 65(3):446–9. 10.1097/00007890-199802150-00030 9484771

[100] Liu X Guo JW Lin XC Tuo YH Peng WL He SY Macrophage NFATc3 prevents foam cell formation and atherosclerosis: evidence and mechanisms. Eur Heart J. (2021) 42(47):4847–61. 10.1093/eurheartj/ehab660 34570211

[101] Ma C Xia R Yang S Liu L Zhang J Feng K Formononetin attenuates atherosclerosis via regulating interaction between KLF4 and SRA in apoE-/- mice. Theranostics. (2020) 10(3):1090–106. 10.7150/thno.38115 31938053 PMC6956811

[102] Erbilgin A Seldin MM Wu X Mehrabian M Zhou Z Qi H Transcription factor Zhx2 deficiency reduces atherosclerosis and promotes macrophage apoptosis in mice. Arterioscler Thromb Vasc Biol. (2018) 38(9):2016–27. 10.1161/ATVBAHA.118.311266 30026271 PMC6202168

[103] Allahverdian S Chaabane C Boukais K Francis GA Bochaton-Piallat ML. Smooth muscle cell fate and plasticity in atherosclerosis. Cardiovasc Res. (2018) 114(4):540–50. 10.1093/cvr/cvy022 29385543 PMC5852505

[104] Gomez D Owens GK. Smooth muscle cell phenotypic switching in atherosclerosis. Cardiovasc Res. (2012) 95(2):156–64. 10.1093/cvr/cvs115 22406749 PMC3388816

[105] Cheng P Wirka RC Shoa Clarke L Zhao Q Kundu R Nguyen T ZEB2 shapes the epigenetic landscape of atherosclerosis. Circulation. (2022) 145(6):469–85. 10.1161/CIRCULATIONAHA.121.057789 34990206 PMC8896308

[106] Lu QB Wan MY Wang PY Zhang CX Xu DY Liao X Chicoric acid prevents PDGF-BB-induced VSMC dedifferentiation, proliferation and migration by suppressing ROS/NFκB/mTOR/P70S6K signaling cascade. Redox Biol. (2018) 14:656–68. 10.1016/j.redox.2017.11.012 29175753 PMC5716955

[107] Ledard N Liboz A Blondeau B Babiak M Moulin C Vallin B Slug, a cancer-related transcription factor, is involved in vascular smooth muscle cell transdifferentiation induced by platelet-derived growth factor-BB during atherosclerosis. J Am Heart Assoc. (2020) 9(2):e014276. 10.1161/JAHA.119.014276 31959031 PMC7033846

[108] Man JJ Beckman JA Jaffe IZ. Sex as a biological variable in atherosclerosis. Circ Res. (2020) 126(9):1297–319. 10.1161/CIRCRESAHA.120.315930 32324497 PMC7185045

[109] Gersh FL O’Keefe JH Lavie CJ. Postmenopausal hormone therapy for cardiovascular health: the evolving data. Heart Br Card Soc. (2021) 107(14):1115–22. 10.1136/heartjnl-2019-316323 33619206

[110] Lee WS Harder JA Yoshizumi M Lee ME Haber E. Progesterone inhibits arterial smooth muscle cell proliferation. Nat Med. (1997) 3(9):1005–8. 10.1038/nm0997-1005 9288727

[111] Karunakaran D Nguyen MA Geoffrion M Vreeken D Lister Z Cheng HS RIPK1 expression associates with inflammation in early atherosclerosis in humans and can be therapeutically silenced to reduce NF-κB activation and atherogenesis in mice. Circulation. (2021) 143(2):163–77. 10.1161/CIRCULATIONAHA.118.038379 33222501

[112] Luo JY Cheng CK He L Pu Y Zhang Y Lin X Endothelial UCP2 is a mechanosensitive suppressor of atherosclerosis. Circ Res. (2022) 131(5):424–41. 10.1161/CIRCRESAHA.122.321187 35899624 PMC9390236

[113] Subramanian V Golledge J Ijaz T Bruemmer D Daugherty A. Pioglitazone-induced reductions in atherosclerosis occur via smooth muscle cell-specific interaction with PPAR{gamma}. Circ Res. (2010) 107(8):953–8. 10.1161/CIRCRESAHA.110.219089 20798360 PMC2963621

[114] Yang HX Zhang M Long SY Tuo QH Tian Y Chen JX Cholesterol in LDL receptor recycling and degradation. Clin Chim Acta Int J Clin Chem. (2020) 500:81–6. 10.1016/j.cca.2019.09.022 31770510

[115] Tao H Yancey PG Blakemore JL Zhang Y Ding L Jerome WG Macrophage SR-BI modulates autophagy via VPS34 complex and PPARα transcription of tfeb in atherosclerosis. J Clin Invest. (2021) 131(7):e94229. 10.1172/JCI94229 33661763 PMC8011903

[116] Hou K Li S Zhang M Qin X. Caveolin-1 in autophagy: a potential therapeutic target in atherosclerosis. Clin Chim Acta Int J Clin Chem. (2021) 513:25–33. 10.1016/j.cca.2020.11.020 33279502

[117] Yuan HY Kuang SY Zheng X Ling HY Yang YB Yan PK Curcumin inhibits cellular cholesterol accumulation by regulating SREBP-1/caveolin-1 signaling pathway in vascular smooth muscle cells. Acta Pharmacol Sin. (2008) 29(5):555–63. 10.1111/j.1745-7254.2008.00783.x 18430363

[118] Edsfeldt A Swart M Singh P Dib L Sun J Cole JE Interferon regulatory factor-5-dependent CD11c+ macrophages contribute to the formation of rupture-prone atherosclerotic plaques. Eur Heart J. (2022) 43(19):1864–77. 10.1093/eurheartj/ehab920 35567557 PMC9113304

[119] Chiu JJ Chien S. Effects of disturbed flow on vascular endothelium: pathophysiological basis and clinical perspectives. Physiol Rev. (2011) 91(1):327–87. 10.1152/physrev.00047.2009 21248169 PMC3844671

[120] Grundtman C Kreutmayer SB Almanzar G Wick MC Wick G. Heat shock protein 60 and immune inflammatory responses in atherosclerosis. Arterioscler Thromb Vasc Biol. (2011) 31(5):960–8. 10.1161/ATVBAHA.110.217877 21508342 PMC3212728

[121] Krishnamurthy K Glaser S Alpini GD Cardounel AJ Liu Z Ilangovan G. Heat shock factor-1 knockout enhances cholesterol 7α-hydroxylase (CYP7A1) and multidrug transporter (MDR1) gene expressions to attenuate atherosclerosis. Cardiovasc Res. (2016) 111(1):74–83. 10.1093/cvr/cvw094 27131506 PMC4909164

[122] Jaiswal S Natarajan P Silver AJ Gibson CJ Bick AG Shvartz E Clonal hematopoiesis and risk of atherosclerotic cardiovascular disease. N Engl J Med. (2017) 377(2):111–21. 10.1056/NEJMoa1701719 28636844 PMC6717509

[123] Fuster JJ MacLauchlan S Zuriaga MA Polackal MN Ostriker AC Chakraborty R Clonal hematopoiesis associated with TET2 deficiency accelerates atherosclerosis development in mice. Science. (2017) 355(6327):842–7. 10.1126/science.aag1381 28104796 PMC5542057

[124] Ampomah PB Cai B Sukka SR Gerlach BD Yurdagul A Wang X Macrophages use apoptotic cell-derived methionine and DNMT3A during efferocytosis to promote tissue resolution. Nat Metab. (2022) 4(4):444–57. 10.1038/s42255-022-00551-7 35361955 PMC9050866

[125] Tyrrell DJ Goldstein DR. Ageing and atherosclerosis: vascular intrinsic and extrinsic factors and potential role of IL-6. Nat Rev Cardiol. (2021) 18(1):58–68. 10.1038/s41569-020-0431-7 32918047 PMC7484613

[126] Morrell NW Adnot S Archer SL Dupuis J Lloyd Jones P MacLean MR Cellular and molecular basis of pulmonary arterial hypertension. J Am Coll Cardiol. (2009) 54(1 Suppl):S20–31. 10.1016/j.jacc.2009.04.018 19555855 PMC2790324

[127] Hassoun PM. Pulmonary arterial hypertension. N Engl J Med. (2021) 385(25):2361–76. 10.1056/NEJMra2000348 34910865

[128] Tuder RM Stacher E Robinson J Kumar R Graham BB. Pathology of pulmonary hypertension. Clin Chest Med. (2013) 34(4):639–50. 10.1016/j.ccm.2013.08.009 24267295

[129] International PPH Consortium, Lane KB Machado RD Pauciulo MW Thomson JR Phillips JA Heterozygous germline mutations in BMPR2, encoding a TGF-beta receptor, cause familial primary pulmonary hypertension. Nat Genet. (2000) 26(1):81–4. 10.1038/79226 10973254

[130] Teichert-Kuliszewska K Kutryk MJB Kuliszewski MA Karoubi G Courtman DW Zucco L Bone morphogenetic protein receptor-2 signaling promotes pulmonary arterial endothelial cell survival: implications for loss-of-function mutations in the pathogenesis of pulmonary hypertension. Circ Res. (2006) 98(2):209–17. 10.1161/01.RES.0000200180.01710.e6 16357305

[131] Alastalo TP Li M Perez V Pham D Sawada H Wang JK Disruption of PPARγ/β-catenin-mediated regulation of apelin impairs BMP-induced mouse and human pulmonary arterial EC survival. J Clin Invest. (2011) 121(9):3735–46. 10.1172/JCI43382 21821917 PMC3163943

[132] Liang OD So EY Egan PC Goldberg LR Aliotta JM Wu KQ Endothelial to haematopoietic transition contributes to pulmonary arterial hypertension. Cardiovasc Res. (2017) 113(13):1560–73. 10.1093/cvr/cvx161 29016733 PMC5852529

[133] Chen MJ Yokomizo T Zeigler BM Dzierzak E Speck NA. Runx1 is required for the endothelial to haematopoietic cell transition but not thereafter. Nature. (2009) 457(7231):887–91. 10.1038/nature07619 19129762 PMC2744041

[134] Thenappan T Ormiston ML Ryan JJ Archer SL. Pulmonary arterial hypertension: pathogenesis and clinical management. Br Med J. (2018) 360:j5492. 10.1136/bmj.j5492 29540357 PMC6889979

[135] Li D Shao NY Moonen JR Zhao Z Shi M Otsuki S ALDH1A3 coordinates metabolism with gene regulation in pulmonary arterial hypertension. Circulation. (2021) 143(21):2074–90. 10.1161/CIRCULATIONAHA.120.048845 33764154 PMC8289565

[136] Steffes LC Froistad AA Andruska A Boehm M McGlynn M Zhang F A Notch3-marked subpopulation of vascular smooth muscle cells is the cell of origin for occlusive pulmonary vascular lesions. Circulation. (2020) 142(16):1545–61. 10.1161/CIRCULATIONAHA.120.045750 32794408 PMC7578108

[137] Chan SY Loscalzo J. Pathogenic mechanisms of pulmonary arterial hypertension. J Mol Cell Cardiol. (2008) 44(1):14–30. 10.1016/j.yjmcc.2007.09.006 17950310 PMC2234575

[138] Stenmark KR Gerasimovskaya E Nemenoff RA Das M. Hypoxic activation of adventitial fibroblasts: role in vascular remodeling. Chest. (2002) 122(6 Suppl):326S–34S. 10.1378/chest.122.6_suppl.326S 12475810

[139] Li Y Zhang L Wang X Chen M Liu Y Xing Y Elk-1-mediated 15-lipoxygenase expression is required for hypoxia-induced pulmonary vascular adventitial fibroblast dynamics. Acta Physiol Oxf Engl. (2016) 218(4):276–89. 10.1111/apha.12711 27174674

[140] Chen C Han X Fan F Liu Y Wang T Wang J Serotonin drives the activation of pulmonary artery adventitial fibroblasts and TGF-β1/Smad3-mediated fibrotic responses through 5-HT(2A) receptors. Mol Cell Biochem. (2014) 397(1–2):267–76. 10.1007/s11010-014-2194-0 25185755

[141] Morrell NW Aldred MA Chung WK Elliott CG Nichols WC Soubrier F Genetics and genomics of pulmonary arterial hypertension. Eur Respir J. (2019) 53(1):1801899. 10.1183/13993003.01899-2018 30545973 PMC6351337

[142] Shimoda LA. Cellular pathways promoting pulmonary vascular remodeling by hypoxia. Physiol Bethesda Md. (2020) 35(4):222–33. 10.1152/physiol.00039.2019 PMC7474258 32490752

[143] Ma L Li G Zhu H Dong X Zhao D Jiang X 2-Methoxyestradiol synergizes with sorafenib to suppress hepatocellular carcinoma by simultaneously dysregulating hypoxia-inducible factor-1 and -2. Cancer Lett. (2014) 355(1):96–105. 10.1016/j.canlet.2014.09.011 25218350

[144] Tofovic SP Jackson EK. Estradiol metabolism: crossroads in pulmonary arterial hypertension. Int J Mol Sci. (2019) 21(1):116. 10.3390/ijms21010116 31877978 PMC6982327

[145] Saadoun D Vautier M Cacoub P. Medium- and large-vessel vasculitis. Circulation. (2021) 143(3):267–82. 10.1161/CIRCULATIONAHA.120.046657 33464968

[146] Langford CA. Vasculitis. J Allergy Clin Immunol. (2010) 125(2 Suppl 2):S216–225. 10.1016/j.jaci.2009.07.002 19932919

[147] Miller A Basu N Luqmani R. Assessment of systemic vasculitis. Autoimmun Rev. (2008) 8(2):170–5. 10.1016/j.autrev.2008.07.001 18672099

[148] Tombetti E Mason JC. Takayasu arteritis: advanced understanding is leading to new horizons. Rheumatol Oxf Engl. (2019) 58(2):206–19. 10.1093/rheumatology/key040 29635396

[149] Le Joncour A Desbois AC Leroyer AS Tellier E Régnier P Maciejewski-Duval A Mast cells drive pathologic vascular lesions in takayasu arteritis. J Allergy Clin Immunol. (2022) 149(1):292–301.e3. 10.1016/j.jaci.2021.05.003 33992671

[150] Régnier P Le Joncour A Maciejewski-Duval A Desbois AC Comarmond C Rosenzwajg M Targeting JAK/STAT pathway in Takayasu’s arteritis. Ann Rheum Dis. (2020) 79(7):951–9. 10.1136/annrheumdis-2019-216900 32213496

[151] Kitching AR Anders HJ Basu N Brouwer E Gordon J Jayne DR ANCA-associated vasculitis. Nat Rev Dis Primer. (2020) 6(1):71. 10.1038/s41572-020-0204-y 32855422

[152] Halbwachs L Lesavre P. Endothelium-neutrophil interactions in ANCA-associated diseases. J Am Soc Nephrol JASN. (2012) 23(9):1449–61. 10.1681/ASN.2012020119 22942199 PMC3431419

[153] Harnden A Tulloh R Burgner D. Kawasaki disease. Br Med J. (2014) 349(Sep17 3):g5336. 10.1136/bmj.g5336 25230954

[154] Noval Rivas M Arditi M. Kawasaki disease: pathophysiology and insights from mouse models. Nat Rev Rheumatol. (2020) 16(7):391–405. 10.1038/s41584-020-0426-0 32457494 PMC7250272

[155] McCrindle BW Rowley AH Newburger JW Burns JC Bolger AF Gewitz M Diagnosis, treatment, and long-term management of kawasaki disease: a scientific statement for health professionals from the American heart association. Circulation. (2017) 135(17):e927–99. 10.1161/CIR.0000000000000484 28356445

[156] Huang H Dong J Jiang J Yang F Zheng Y Wang S The role of FOXO4/NFAT2 signaling pathway in dysfunction of human coronary endothelial cells and inflammatory infiltration of vasculitis in kawasaki disease. Front Immunol. (2022) 13:1090056. 10.3389/fimmu.2022.1090056 36700213 PMC9869249

[157] Ting HA Schaller MA de Almeida Nagata DE Rasky AJ Maillard IP Lukacs NW. Notch ligand Delta-like 4 promotes regulatory T cell identity in pulmonary viral infection. J Immunol Baltim Md 1950. (2017) 198(4):1492–502. 10.4049/jimmunol.1601654 PMC5296281 28077598

[158] Hoffman GS. Giant cell arteritis. Ann Intern Med. (2016) 165(9):ITC65–80. 10.7326/AITC201611010 27802475

[159] Jin K Wen Z Wu B Zhang H Qiu J Wang Y NOTCH-induced rerouting of endosomal trafficking disables regulatory T cells in vasculitis. J Clin Invest. (2021) 131(1):e136042. 10.1172/JCI136042 32960812 PMC7773364

[160] Sato Y Jain A Ohtsuki S Okuyama H Sturmlechner I Takashima Y Stem-like CD4+ T cells in perivascular tertiary lymphoid structures sustain autoimmune vasculitis. Sci Transl Med. (2023) 15(712):eadh0380. 10.1126/scitranslmed.adh0380 37672564 PMC11131576

[161] Amadi-Obi A Yu CR Liu X Mahdi RM Clarke GL Nussenblatt RB TH17 Cells contribute to uveitis and scleritis and are expanded by IL-2 and inhibited by IL-27/STAT1. Nat Med. (2007) 13(6):711–8. 10.1038/nm1585 17496900

[162] Yang Y Wang Q Xie M Liu P Qi X Liu X Berberine exerts an anti-inflammatory role in ocular behcet’s disease. Mol Med Rep. (2017) 15(1):97–102. 10.3892/mmr.2016.5980 27922688 PMC5355754

[163] Solis AG Bielecki P Steach HR Sharma L Harman CCD Yun S Mechanosensation of cyclical force by PIEZO1 is essential for innate immunity. Nature. (2019) 573(7772):69–74. 10.1038/s41586-019-1485-8 31435009 PMC6939392

[164] Deplancke B Alpern D Gardeux V. The genetics of transcription factor DNA binding variation. Cell. (2016) 166(3):538–54. 10.1016/j.cell.2016.07.012 27471964

[165] Wang D Wu J Le S Wang H Luo J Li R Oltipraz, the activator of nuclear factor erythroid 2-related factor 2 (Nrf2), protects against the formation of BAPN-induced aneurysms and dissection of the thoracic aorta in mice by inhibiting activation of the ROS-mediated NLRP3 inflammasome. Eur J Pharmacol. (2022) 936:175361. 10.1016/j.ejphar.2022.175361 36336010

[166] Li Y Tao L Xu Y Guo R. Taxifolin ameliorates abdominal aortic aneurysm by preventing inflammation and apoptosis and extracellular matrix degradation via inactivating TLR4/NF-κB axis. Int Immunopharmacol. (2023) 119:110197. 10.1016/j.intimp.2023.110197 37098322

[167] Yao F Yao Z Zhong T Zhang J Wang T Zhang B Imatinib prevents elastase-induced abdominal aortic aneurysm progression by regulating macrophage-derived MMP9. Eur J Pharmacol. (2019) 860:172559. 10.1016/j.ejphar.2019.172559 31325435

[168] Que Y Shu X Wang L Wang S Li S Hu P Inactivation of SERCA2 Cys674 accelerates aortic aneurysms by suppressing PPARγ. Br J Pharmacol. (2021) 178(11):2305–23. 10.1111/bph.15411 33591571

[169] Ding X Zheng L Yang B Wang X Ying Y. Luteolin attenuates atherosclerosis via modulating signal transducer and activator of transcription 3-mediated inflammatory response. Drug Des Devel Ther. (2019) 13:3899–911. 10.2147/DDDT.S207185 31819365 PMC6874161

[170] Guo Y Fan Y Zhang J Lomberk GA Zhou Z Sun L Perhexiline activates KLF14 and reduces atherosclerosis by modulating ApoA-I production. J Clin Invest. (2015) 125(10):3819–30. 10.1172/JCI79048 26368306 PMC4607137

[171] Li H Mani S Wu L Fu M Shuang T Xu C The interaction of estrogen and CSE/H2S pathway in the development of atherosclerosis. Am J Physiol Heart Circ Physiol. (2017) 312(3):H406–14. 10.1152/ajpheart.00245.2016 27986657

[172] Evans TD Jeong SJ Zhang X Sergin I Razani B. TFEB and trehalose drive the macrophage autophagy-lysosome system to protect against atherosclerosis. Autophagy. (2018) 14(4):724–6. 10.1080/15548627.2018.1434373 29394113 PMC5959328

[173] Zetterqvist AV Berglund LM Blanco F Garcia-Vaz E Wigren M Dunér P Inhibition of nuclear factor of activated T-cells (NFAT) suppresses accelerated atherosclerosis in diabetic mice. PloS One. (2014) 8(6):e65020. 10.1371/journal.pone.0065020 23755169 PMC3670844

[174] Lei W Deng YF Hu XY Ni JN Jiang M Bai G. Phthalides, senkyunolide A and ligustilide, show immunomodulatory effect in improving atherosclerosis, through inhibiting AP-1 and NF-κB expression. Biomed Pharmacother Biomedecine Pharmacother. (2019) 117:109074. 10.1016/j.biopha.2019.109074 31177061

[175] Cai D Liu H Wang J Hou Y Pang T Lin H Balasubramide derivative 3C attenuates atherosclerosis in apolipoprotein E-deficient mice: role of AMPK-STAT1-STING signaling pathway. Aging. (2021) 13(8):12160–78. 10.18632/aging.202929 33901014 PMC8109080

[176] Shahid S Pantakani M Binder L Fischer A Pantakani K Asif AR. Small molecule BRD4 inhibitors apabetalone and JQ1 rescues endothelial cells dysfunction, protects monolayer integrity and reduces midkine expression. Mol Basel Switz. (2022) 27(21):7453. 10.3390/molecules27217453 PMC9692972 36364277

[177] Pan H Guo Z Lv P Hu K Wu T Lin Z Proline/serine-rich coiled-coil protein 1 inhibits macrophage inflammation and delays atherosclerotic progression by binding to annexin A2. Clin Transl Med. (2023) 13(3):e1220. 10.1002/ctm2.1220 36932468 PMC10023832

[178] Liu Y Wang X Pang J Zhang H Luo J Qian X Attenuation of atherosclerosis by protocatechuic acid via inhibition of M1 and promotion of M2 macrophage polarization. J Agric Food Chem. (2019) 67(3):807–18. 10.1021/acs.jafc.8b05719 30592218

[179] Fan P Meng H Hao W Zheng Y Li H Zhang Z Cardamonin targets KEAP1/NRF2 signaling for protection against atherosclerosis. Food Funct. (2023) 14(10):4905–20. 10.1039/D3FO00967J 37157847

[180] Dai Z Zhu MM Peng Y Machireddy N Evans CE Machado R Therapeutic targeting of vascular remodeling and right heart failure in pulmonary arterial hypertension with a HIF-2α inhibitor. Am J Respir Crit Care Med. (2018) 198(11):1423–34. 10.1164/rccm.201710-2079OC 29924941 PMC6290950

[181] Kishimoto Y Kato T Ito M Azuma Y Fukasawa Y Ohno K Hydrogen ameliorates pulmonary hypertension in rats by anti-inflammatory and antioxidant effects. J Thorac Cardiovasc Surg. (2015) 150(3):645–54.e3. 10.1016/j.jtcvs.2015.05.052 26095621

[182] Zhang M Wu Y Wang M Wang Y Tausif R Yang Y. Genistein rescues hypoxia-induced pulmonary arterial hypertension through estrogen receptor and β-adrenoceptor signaling. J Nutr Biochem. (2018) 58:110–8. 10.1016/j.jnutbio.2018.04.016 29886191

[183] Zhang B Niu W Xu D Li Y Liu M Wang Y Oxymatrine prevents hypoxia- and monocrotaline-induced pulmonary hypertension in rats. Free Radic Biol Med. (2014) 69:198–207. 10.1016/j.freeradbiomed.2014.01.013 24440469

[184] Chuang KH Yao RH Jiang YN Gui LX Zheng SY Lin MJ. Attenuating effect of magnesium on pulmonary arterial calcification in rodent models of pulmonary hypertension. J Hypertens. (2022) 40(10):1979–93. 10.1097/HJH.0000000000003211 36052522

[185] Jiang W Sun M Wang Y Zheng M Yuan Z Mai S Critical role of notch-1 in mechanistic target of rapamycin hyperactivity and vascular inflammation in patients with takayasu arteritis. Arthritis Rheumatol Hoboken NJ. (2022) 74(7):1235–44. 10.1002/art.42103 35212196

[186] Cui X Kong X Chen R Ma L Jiang L. The potential role of leflunomide in inhibiting vascular fibrosis by down-regulating type-II macrophages in takayasu’s arteritis. Clin Exp Rheumatol. (2020) 38(2):69–78. 31969231

[187] Ding Y Peng Y Wu H Huang Y Sheng K Li C The protective roles of liraglutide on kawasaki disease via AMPK/mTOR/NF-κB pathway. Int Immunopharmacol. (2023) 117:110028. 10.1016/j.intimp.2023.110028 36934674

[188] Merkle M Pircher J Mannell H Krötz F Blüm P Czermak T LL37 Inhibits the inflammatory endothelial response induced by viral or endogenous DNA. J Autoimmun. (2015) 65:19–29. 10.1016/j.jaut.2015.07.015 26297208

[189] Henley MJ Koehler AN. Advances in targeting “undruggable” transcription factors with small molecules. Nat Rev Drug Discov. (2021) 20(9):669–88. 10.1038/s41573-021-00199-0 34006959

[190] Joung J Ma S Tay T Geiger-Schuller KR Kirchgatterer PC Verdine VK A transcription factor atlas of directed differentiation. Cell. (2023) 186(1):209–29.e26. 10.1016/j.cell.2022.11.026 36608654 PMC10344468

[191] Matys V Kel-Margoulis OV Fricke E Liebich I Land S Barre-Dirrie A TRANSFAC and its module TRANSCompel: transcriptional gene regulation in eukaryotes. Nucleic Acids Res. (2006) 34(Database issue):D108–110. 10.1093/nar/gkj143 16381825 PMC1347505

[192] Mathelier A Fornes O Arenillas DJ Chen CY Denay G Lee J JASPAR 2016: a major expansion and update of the open-access database of transcription factor binding profiles. Nucleic Acids Res. (2016) 44(D1):D110–115. 10.1093/nar/gkv1176 26531826 PMC4702842

[193] Soto LF Li Z Santoso CS Berenson A Ho I Shen VX Compendium of human transcription factor effector domains. Mol Cell. (2022) 82(3):514–26. 10.1016/j.molcel.2021.11.007 34863368 PMC8818021

[194] DelRosso N Tycko J Suzuki P Andrews C Aradhana N Mukund A Large-scale mapping and mutagenesis of human transcriptional effector domains. Nature. (2023) 616(7956):365–72. 10.1038/s41586-023-05906-y 37020022 PMC10484233

[195] Yang Y Gomez N Infarinato N Adam RC Sribour M Baek I The pioneer factor SOX9 competes for epigenetic factors to switch stem cell fates. Nat Cell Biol. (2023) 25(8):1185–95. 10.1038/s41556-023-01184-y 37488435 PMC10415178

[196] O’Connell DJ Kolde R Sooknah M Graham DB Sundberg TB Latorre I Simultaneous pathway activity inference and gene expression analysis using RNA sequencing. Cell Syst. (2016) 2(5):323–34. 10.1016/j.cels.2016.04.011 27211859 PMC5032147

